# Brachyury‐Activated Fucoidan Hydrogel Microspheres Rejuvenate Degenerative Intervertebral Discs Microenvironment

**DOI:** 10.1002/advs.202504195

**Published:** 2025-06-20

**Authors:** Yuhao Gong, Wenxiao Shi, Xingzhu Liu, Hang Yu, Yinghui Wu, Yanzhang Xia, Caichun Yue, Chongkai Yang, Cong Shen, Renjun Pei, Tianwen Xin, Hailong Pei, Jun Shen

**Affiliations:** ^1^ Department of Orthopedics the Affiliated Suzhou Hospital of Nanjing Medical University Suzhou Municipal Hospital Gusu School Nanjing Medical University Suzhou 215006 P. R. China; ^2^ Suzhou Key Laboratory of Orthopedic Medical Engineering the Affiliated Suzhou Hospital of Nanjing Medical University，Suzhou Municipal Hospital Suzhou 215006 P. R. China; ^3^ CAS Key Laboratory of Nano‐Bio Interface Division of Nanobio‐medicine Suzhou Institute of Nano‐Tech and Nano‐Bionics (SINANO) Chinese Academy of Sciences Suzhou 215123 P. R. China; ^4^ State Key Laboratory of Reproductive Medicine Center for Reproduction and Genetics Suzhou Municipal Hospital The Affiliated Suzhou Hospital of Nanjing Medical University Gusu School Nanjing Medical University Suzhou 215002 P. R. China; ^5^ State Key Laboratory of Radiation Medicine and Protection School of Radiation Medicine and Protection Suzhou Medical College of Soochow University Suzhou Jiangsu 215123 P. R. China

**Keywords:** brachyury, fucoidan, hydrogel microspheres, intervertebral disc degeneration, LNP

## Abstract

Extracellular matrix (ECM) metabolic disorders and the establishment of inflammatory microenvironment are the primary pathological alterations associated with intervertebral disc degeneration (IVDD). The inflammatory microenvironment promotes ECM degradation, further exacerbating the vicious cycle of nucleus pulposus (NP) degeneration. This study introduces the mRNA encoding a novel therapeutic transcription factor, Brachyury (Bry), into nucleus pulposus cells (NPCs) using an injectable microsphere system composed of biomimetic GelMA/Fucoidan (FU) dual‐component hydrogel (GF) and surface chemically grafted lipid nanoparticles (LNP) (BLNP@GF). The study aims to alleviate inflammatory response in the NP while restoring the ECM secretion function of NPCs and enhancing the ability of NPCs to withstand inflammatory stress, thereby restoring physiological balance in the NP microenvironment. The GF microspheres demonstrate injectability and porosity, facilitating efficient LNP loading through chemical grafting. In the LPS‐simulated inflammatory microenvironment, sustained release of FU significantly reduces inflammatory activity in NPCs. Successful transfection with Bry mRNA upregulate ECM synthesis in degenerated NPCs. In a rat tail puncture IVDD model, local application of BLNP@GF microspheres effectively improved ECM remodeling in NP tissue, thereby ameliorating puncture‐induced IVDD. In conclusion, FU‐functionalized GelMA hydrogel microspheres loaded with Bry mRNA provide a promising new strategy for reversing IVDD.

## Graphical abstract

This study describes the creation of injectable Brachyury (Bry) mRNA‐loaded Fucoidan‐functionalized GelMA hydrogel microspheres (BLNP@GF). These microspheres efficiently suppress inflammation, enhance extracellular matrix (ECM) synthesis in degenerated nucleus pulposus cells, and mitigate intervertebral disc degeneration (IVDD) in rat models. By rejuvenating the physiological equilibrium of the disc microenvironment, this innovative approach offers a promising strategy to alleviate IVDD.

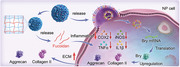

## 1 Introduction

Low back pain (LBP) is a prevalent condition encountered in spinal surgery clinics, which can significantly impact the life quality of patients even in mild cases and potentially result in disability in severe cases.^[^ ^1^ ^]^ With the aggravation of population aging, LBP is causing increasingly serious social and economic burdens globally.^[^ ^2^ ^]^ Intervertebral disc degeneration (IVDD) is a primary etiology underlying LBP and serves as the foundation for various disc‐related disorders. Traditional therapeutic approaches for LBP caused by IVDD disease primarily encompass systemic and supportive therapies, such as self‐care and non‐pharmacological physical therapy.^[^ ^3^, ^4^ ^]^ However, these treatments are unlikely to effectively prevent the progression of IVDD. Consequently, a subset of patients remains inevitably subjected to invasive interventions.^[^ ^5^ ^]^ However, invasive treatments such as surgery cannot restore the biomechanical function of intervertebral discs (IVD), and existing surgical methods may not be suitable for all patients.^[^ ^6^ ^]^ Therefore, it is imperative to urgently address the clinical challenge of effectively alleviating discogenic LBP through prevention or reversal of IVDD while limiting disease progression.

How to reverse early IVDD thereby restoring the extracellular matrix (ECM) environment of nucleus pulposus (NP) by reshaping the physiological metabolism and vitality of NP cells (NPCs) is the major challenge. As a part of the spine, IVD originates from mesodermal tissue during development. Extensive research demonstrated that the transcription factor Brachyury (Bry) played a crucial role in mesodermal development and promoting spinal formation.^[^ ^7^ ^]^ Our previous studies confirmed Bry could promote the synthesis of collagen II and glycosaminoglycans in degenerative NPCs.^[^ ^8^, ^9^ ^]^ However, it remains a challenge to maintain the drug activity of transcription factors and to efficiently act on NPCs when used in vivo.^[^ ^10^ ^]^ Compared to recombinant proteins, mRNA therapy addressed issues of improper protein folding and high costs associated with protein purification.^[^ ^11^ ^]^ The optimization of the 5′‐and 3′‐TR regions in mRNA could enhance translation efficacy, thereby improving therapeutic efficiency.^[^ ^12^, ^13^ ^]^ Among numerous types of injectable carriers with encapsulation capabilities,^[^ ^14^ ^]^ the emerging lipid nanoparticles (LNP) are increasingly garnering widespread attention. LNP is a kind of lipid vesicle composed of a uniform lipid core, used for delivering drugs (such as nucleic acid drugs and gene therapy drugs) to target cells or tissues.^[^ ^15^ ^]^ Naked mRNA is inherently unstable and can be rapidly degraded and autolyzed by nucleases. Encapsulating mRNA within LNP protects it from exogenous ribonucleases while facilitating intracellular mRNA delivery.^[^ ^16^, ^17^ ^]^ Although LNP serves as an excellent carrier for injectable formulations, its application in treating IVDD has not yet been reported. LNP loaded with Bry mRNA (BLNP) hold promise as an effective therapeutic strategy for degenerative NPCs. Given the unique physiological structure of IVD,^[^ ^18^ ^]^ systemic delivery methods yield low efficacy in targeting degenerated IVD, particularly within the NP interior, thus making local injection therapy a focal point for current research endeavors.

To counteract the degenerative alterations in NP tissues, it is imperative not only to enhance the efficiency of NPCs in synthesizing ECM, but also to address the regulation of local inflammatory microenvironment. Excessive spinal loading can precipitate loss of ECM integrity and metabolic disturbances within the NP, leading to upregulation of pro‐inflammatory gene expression. This further activates immune cells such as macrophages, T cells, and lymphocytes present in NP microenvironment.^[^ ^19^ ^]^ The inflammatory microenvironment can directly induce hypersensitization of endogenous sensory neurons and promote the growth of new sensory fibers, thereby precipitating discogenic pain.^[^ ^20^ ^]^ Concurrently, this inflammatory environment adversely affects the normal physiological functions of NPCs. Consequently, identifying suitable and effective tissue engineering materials to replace the NP structure and thus modulate the immune microenvironment in degenerative regions through immune regulation represents a promising strategy for restoring IVD structure and function.^[^ ^21^ ^]^ Fucoidan (FU) is a sulfated anionic polysaccharide derived from marine plants, characterized by a sulfate group structure similar to that of glycosaminoglycans found in the ECM of NP.^[^ ^22^ ^]^ Recent studies reported the application of FU in the treatment of inflammatory diseases associated with the musculoskeletal system, including rheumatoid arthritis (RA) and osteoarthritis (OA).^[^ ^23^, ^24^ ^]^ However, no available literature investigated whether FU directly affects the resistance of NPCs within the inflammatory microenvironment. We hypothesized that localized administration of FU might help restore immune microenvironment balance within early‐stage degenerative NP tissue. However, as a polysaccharide with loose structures, FU exhibits a rapid degradation rate when administered alone via injection treatment, posing challenges to achieving sustained local immune regulatory effects. In recent years, the advent of composite hydrogel microspheres has emerged as a promising solution to this challenge.^[^ ^25^, ^26^, ^27^ ^]^ GelMA (GM) microspheres exhibit exceptional biocompatibility and biosecurity, and their rheological properties post‐curing closely resemble those of NP.^[^ ^28^, ^29^, ^30^ ^]^ Therefore, we proposed to prepare GelMA/FU (GF) microspheres by adding a certain mass of FU to GelMA solution to establish an interpenetrating network carrier, aiming to accomplish this objective. Additionally, given the potential concerns of excessive diffusion and rapid degradation in local tissues associated with the sole application of BLNP injections, we integrated BLNP and GF microspheres (BLNP@GF) via a chemical grafting method.

**Scheme 1:**
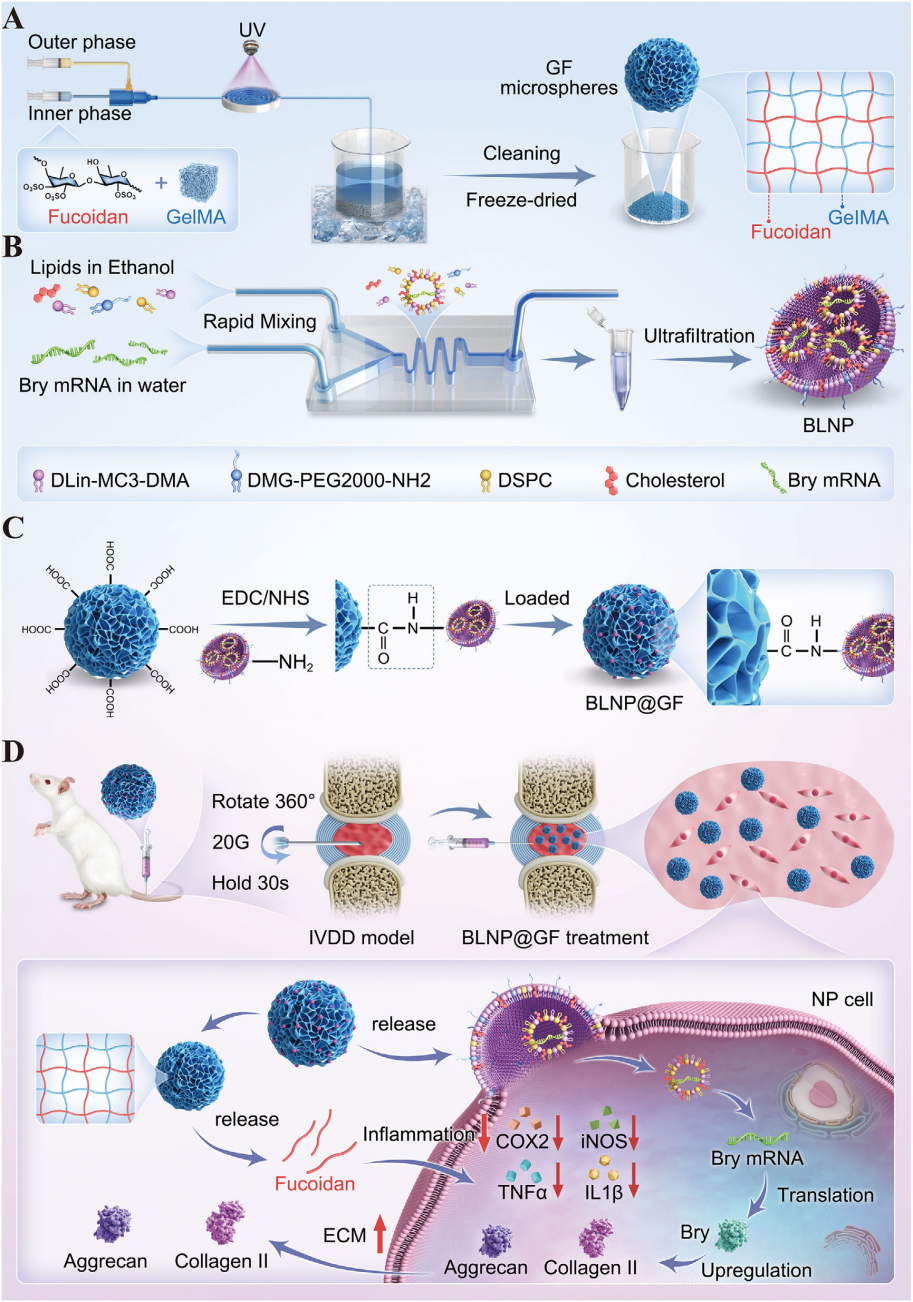
Design of BLNP@GF microspheres to relieve inflammation and promote ECM regeneration A,B,C) Preparation of GF, BLNP and BLNP@GF. D) In situ injection of BLNP@GF microspheres into rat IVDD model for inflammation regulation and ECM regeneration. (GF, GelMA/Fucoidan dual‐component hydrogel; Bry, Brachyury; BLNP, lipid nanoparticles loaded with Bry mRNA; BLNP@GF, GelMA/Fucoidan dual‐component hydrogel microspheres chemically grafted lipid nanoparticles loaded with Bry mRNA; IVDD, intervertebral disc degeneration; ECM, extracellular matrix; NP, nucleus pulposus.).

In this study, microfluidic control was employed to fabricate GF double‐network injectable microspheres and BLNP was chemically grafted onto the GF microspheres via amide bonds, achieving excellent controlled release properties and injectability. The composite BLNP@GF microspheres could modulate inflammatory microenvironment, and further achieve physiological functional repairment through Bry mRNA transfection for NPCs, promoting the reconstruction of ECM physiological environment. In summary, we developed a simple yet effective approach to promote the repairment of NP microenvironment thus reversing the early IVDD process, and providing valuable design insights for delaying the progression of IVDD. (**Scheme** **1**).

## 2 Results

### 2.1 Reduced Expression of Bry in Degenerated Human NPCs and Increased ECM Synthesis with Overexpressed Bry in Degenerative Rat NPCs

**Figure 1:**
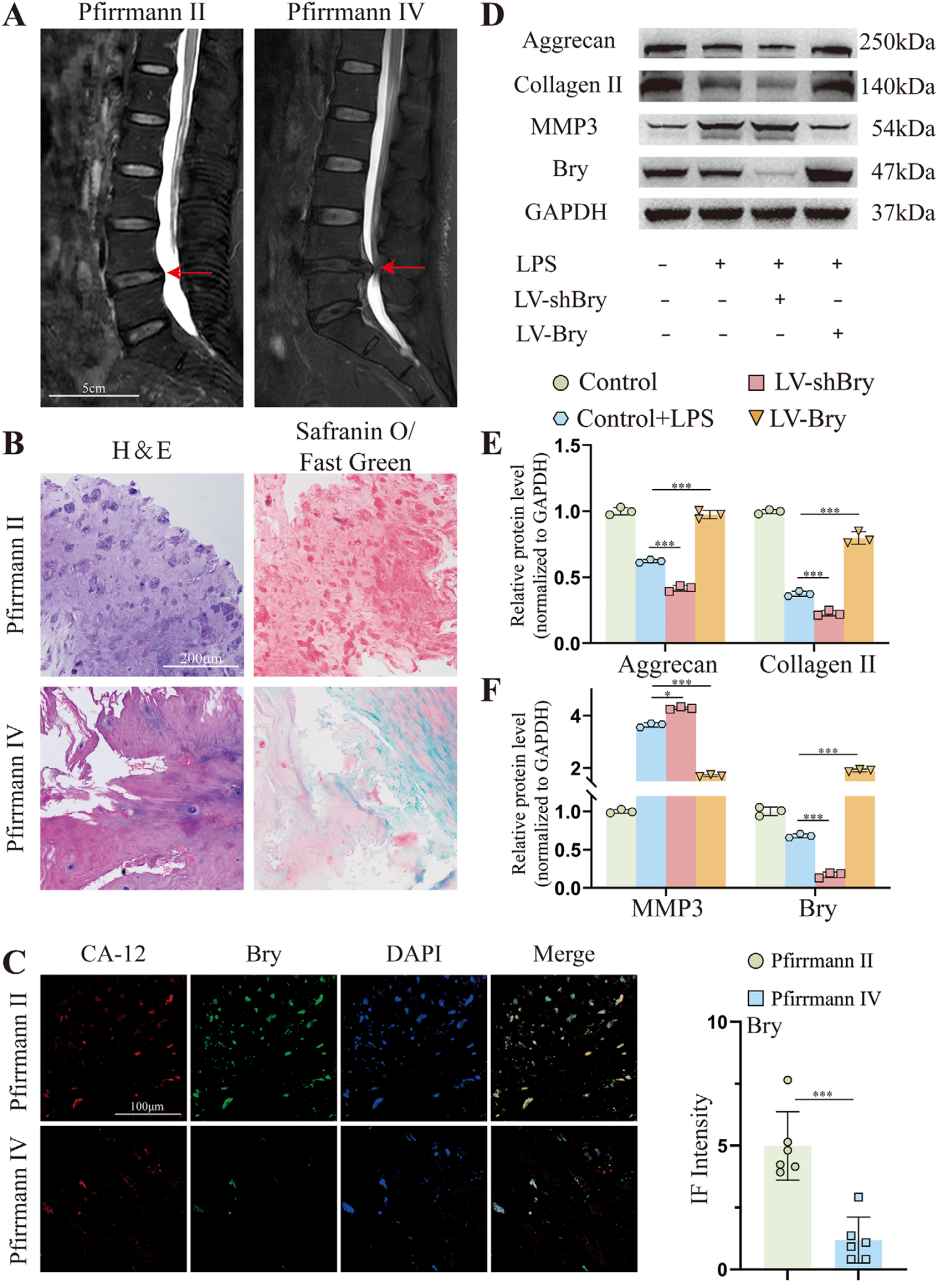
Low expression of Brachyury Bry) in human degenerative nucleus pulposus (NP) tissue. A) T2‐weighted MRI scans showing IVD classified as Pfirrmann grade II and IV (n = 3, red arrows highlight the respective IVDD regions, scale bar = 5 cm). B) Histological evaluation of human NP tissues using H&E and safranin‐O/fast green staining (scale bar = 200 µm). C) Immunofluorescence analysis of CA‐12 and Bry in human NP tissues, and semi‐quantitative analysis of the immunofluorescent staining of Bry in human NP cells. (n = 3, scale bar = 100 µm). D) Western blotting of aggrecan, collagen II, MMP3 and Bry expression in rat NPCs transfected with lentivirus shBry or lentivirus Bry (MOI = 100) and treated with or without LPS (20 ug mL^−1^) for 3 days. E) and F) Semi‐quantitative analysis of aggrecan, collagen II, MMP3 and Bry protein expression (n = 3). All data were presented as mean ± standard deviation, *p < 0.05; **p < 0.01; ***p < 0.001; ns, not significant (one‐way or two‐way ANOVA and Tukey's test compared with each group). MRI, magnetic resonance image; IVD, intervertebral discs; IVDD, intervertebral disc degeneration; H&E, hematoxylin and eosin; CA‐12, carbonic anhydrase‐12.

To evaluate the expression levels of Bry in human NP tissues, we collected NP tissue samples from clinical cases categorized as Pfirrmann grade II and grade IV (n = 3 for each group). As **Figure** **1**A showed, spinal MRI images of IVDD patients were performed, and analysis of T2‐weighted MRI images revealed that the signal intensity in the grade IV group was significantly decreased compared to the grade II group. In addition, H&E and Safranin‐O/fast green staining revealed reduced NPC cellularity and ECM content within the grade IV group (Figure 1B). Carbonic anhydrase‐12 (CA‐12), a classic NP cell marker, when co‐stained with Bry, showed that the fluorescence intensity of Bry was 76.2% lower in NPCs of the grade IV group compared to the grade II group (Figure 1C). These results demonstrated a marked reduction of Bry expression in degenerative NP tissues. Consequently, we verified whether Bry played an important role in degenerative NPCs. Our previous study^[^ ^8^ ^]^ showed that NPCs degeneration was induced by exposure to 20 µg mL^−1^ LPS for 72 h. The WB results showed that, in LPS‐treated NPCs, knockdown of Bry expression led to a reduction in ECM expression and an increase in MMP3 expression, while overexpression of Bry had the opposite effect (Figure 1D–F). These findings underscored the sufficient effect of Bry on IVDD and the pressing need for an effective delivery method to elevate Bry expression levels for alleviating IVDD.

### 2.2 Preparation and Characterization of GF Microspheres

**Figure 2:**
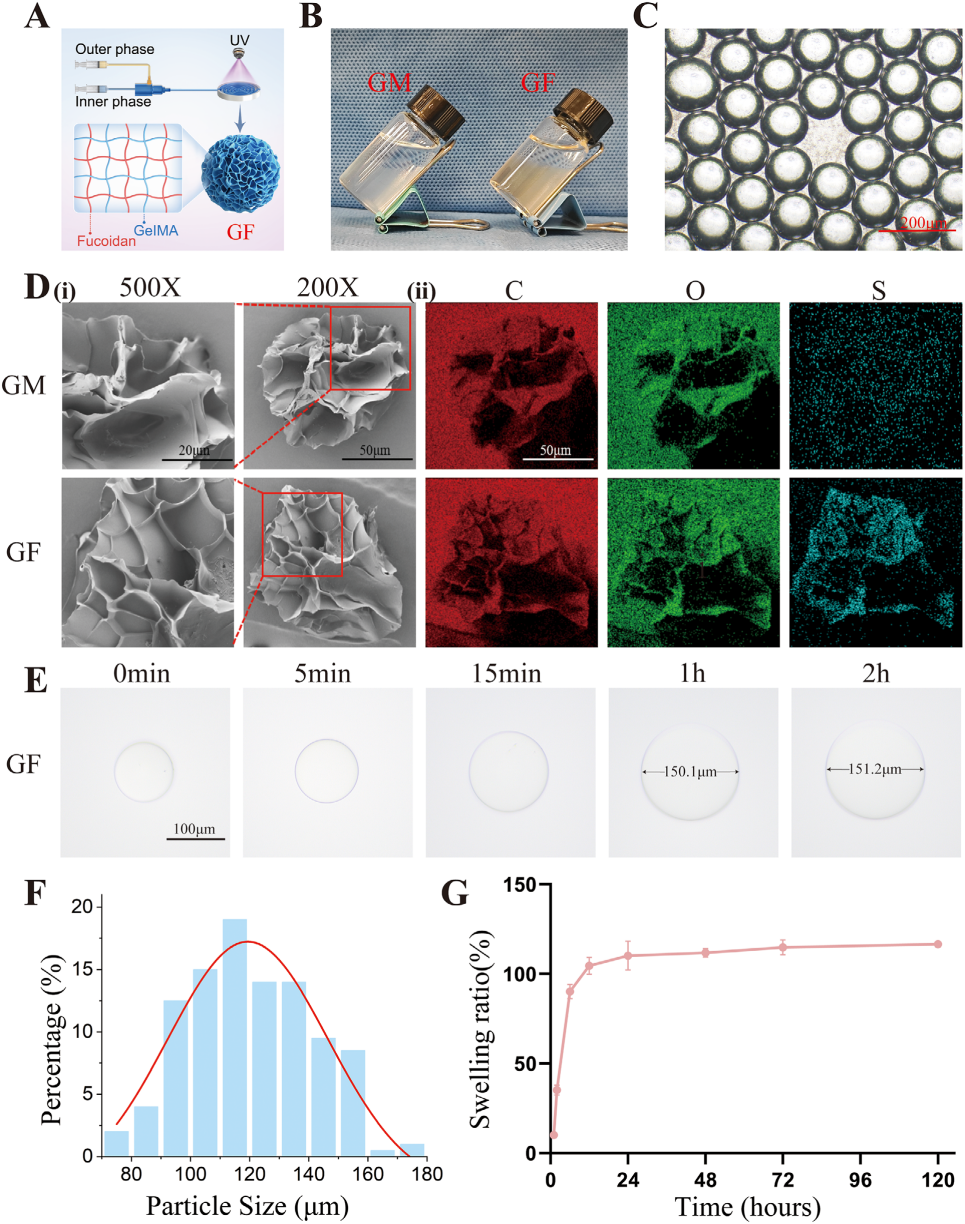
Preparation and characterization of GM and GF microspheres. A) Schematic diagram of the preparation of GF microspheres. B) Images of GM and GF microspheres. C) Morphology of GF microspheres under a brightfield microscopy (scale bar = 200 µm). D) (i) Morphology of GM and GF microspheres under a scanning electron microscope (SEM) (500X, scale bar = 20 µm, 200X, scale bar = 50 µm); (ii) Energy dispersive spectroscopy (EDS) analysis of GM and GF microspheres (scale bar = 50 µm). E) The swelling volume changes of GF microspheres in PBS under a brightfield microscopy. (scale bar = 100 µm) F) Particle size analysis of GF microspheres. G) Swelling ratio of GF microspheres in 120 h.

GM microspheres are carriers with excellent biocompatibility and drug‐loading capacity. The GM microspheres used in this study were prepared using microfluidics. Their main stable solid‐state structures were formed through photo‐crosslinking GM hydrogel (**Figure** **2**A). On this basis, we also evaluated the effect of FU incorporation on the morphology and physicochemical properties of hydrogel microspheres. The morphology of GM and GF microspheres was basically the same when observed directly, showing a uniform and tiny translucent spherical shape. GF microspheres had an overall brown color due to the addition of FU (Figure 2B). Under bright‐field microscopy, the GM microspheres exhibited a uniformly consistent translucent spherical shape (Figure 2C). Analysis of the particle size distribution indicated that the majority of the GM microspheres had diameters ranging between 100 to 120 µm (Figure 2F). After the freeze‐drying, GM and GF microspheres were observed using SEM (Figure 2D(i)). SEM images showed that both GM and GF microspheres presented a uniform porous spherical structure, and there was no obvious difference in size. To further verify the successful fabrication of the microspheres, GM and GF microspheres were analyzed using EDS (Figure 2D(ii)). Carbon (C) and Oxygen (O) were the basic elements of GM. In contrast to GM, the Sulfur (S) element was detected in the GF in addition to C and O. These results demonstrated that the FU with the sulfuric acid group was loaded. The results of the swelling performance of the microspheres showed that the volume of GF microspheres rapidly swelled by 50% within 1 h (Figure 2E) and reached swelling equilibrium within 24 h, with a swelling rate of more than 100% (Figure 2G). It was proved that GF microspheres still retained the good swelling properties of hydrogel microspheres. All in all, a FU‐loaded microsphere vector was successfully prepared, laying the foundation for the delivery of Bry.

### 2.3 Preparation and Characterization of LNP, BLNP and BLNP@GF Microspheres

**Figure 3:**
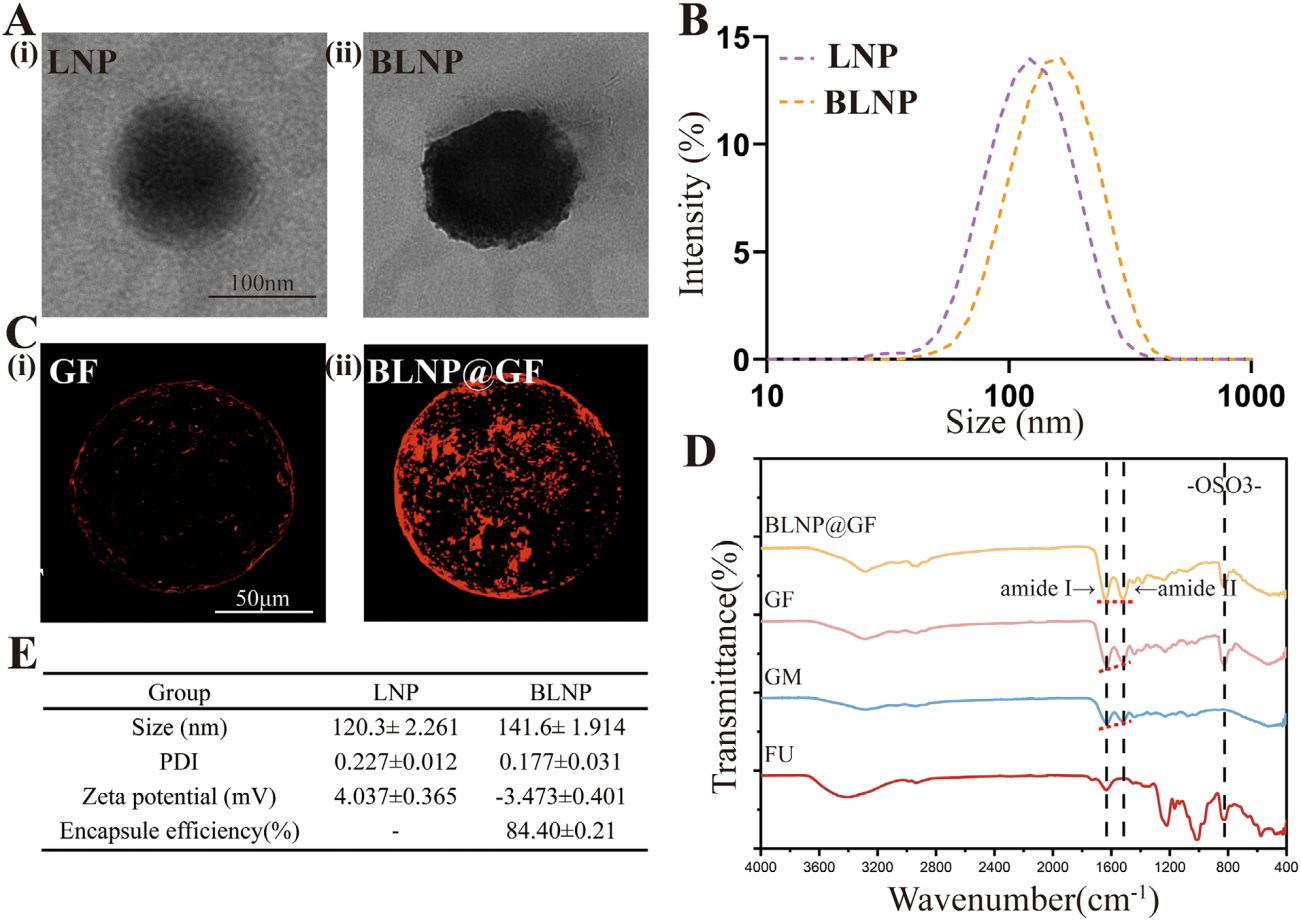
Preparation and characterization of LNP, BLNP, and BLNP@GF. A) Morphology of LNP and BLNP under transmission electron microscopy (TEM) (scale bar = 100 µm). B) Particle size analysis of LNP and BLNP. C) Dil staining of GF and BLNP@GF microspheres (scale bar = 50 µm). D) Fourier‐transform infrared analysis (FTIR) of FU, GM, GF and BLNP@GF. The red dashed lines represent the comparison of the peak sizes of the amide I bond and the amide II bond. E) Size, PDI, Zeta potential and Encapsule efficiency analysis of LNP and BLNP.

To achieve efficient transfection of Bry mRNA into NPCs, this study employed LNP as the carrier for mRNA and developed BLNP. Subsequently, BLNP was chemically grafted onto the surface of GF microspheres to facilitate the delivery of Bry mRNA. We used TEM to observe empty LNP and BLNP (**Figure** **3**A). The TEM images showed that LNP had a bilayer membrane structure with a diameter of about 120 nm. BLNP exhibited deeper staining and enhanced opacity, possibly due to the encapsulation of Bry mRNA within the LNP. The particle size of LNP was 120.3 ± 2.261 nm and the particle size of BLNP was 141.6 ± 1.914 nm (Figure 3B). LNP exhibited a Zeta potential of 4.037 ± 0.365 mV, while BLNP showed a Zeta potential of −3.473 ± 0.401 mV. Notably, the zeta potential of BLNP became negative compared with LNP. This was due to the negative charge carried by Bry mRNA neutralizing the positive charge of the LNP, causing BLNP to appear negatively charged. The encapsulation efficiency, calculated following a previous research method,^[^ ^31^ ^]^ was (84.40 ± 0.21) % (Figure 3E). These above results proved that the Bry mRNA was successfully encapsulated in LNP.

To determine whether BLNP was successfully grafted onto the GF microspheres, we utilized the Dil staining kit. Dil is a lipophilic phospholipid membrane dye capable of binding to the lipids within the BLNP. Fluorescence imaging revealed that the surface of the BLNP@GF microspheres exhibited strong red fluorescence, whereas the GF microspheres showed almost no luminescence. (Figure 3C) Based on these observations, we could conclude that BLNP was successfully conjugated to the surface of the GF microspheres.

The FTIR results showed that a significant vibration band at 830 cm^−1^ was observed in the FU group, which was used to identify the position of the sulfate group (‐OSO3‐) on the fucose monosaccharide unit. Extracting this structural information from FTIR was an effective proof method to characterize the structure and potential function of FU extracts.^[^ ^32^ ^]^ At the same time, the bands at ≈1220–1250 and 1016 cm^−1^ could also be assigned to C‐O‐S and S‐O vibrations, but it was challenging to explicitly assign the chemical bond composition of these bands. Therefore, in this experiment, the most characteristic ‐OSO3‐ absorption peak at 830 cm^−1^ was used to confirm the presence of FU. The amide I band caused by C = O expansion vibration was observed at 1630 cm^−1^ and the amide II band caused by N‐H bending vibration and C‐N expansion vibration at 1520 cm^−1^ in the infrared spectra of both GM and GF group, and the ‐OSO3‐ absorption peak was still observed at 830 cm^−1^ in the GF group, but this phenomenon could not be observed in the GM group, which proved that FU was successfully loaded in GF microspheres. Compared with the GF group, the amide II band's strength of BLNP@GF group increased, and the ratio of the amide II band to the amide I band increased (Figure 3D). It was proved that the dehydration‐condensation reaction of ‐COOH on the surface of the GF microspheres and ‐NH2 on the surface of LNP through NHS/EDC activation caused stronger N‐H bending vibration and C‐N expansion vibration, and LNP was successfully grafted on the surface of the GF microspheres by amide bonds.^[^ ^33^ ^]^ As shown in Figure S1, Supporting Information, BLNP@GF had good injectability, which ensured that BLNP@GF could be used for injectable treatment in the IVDD.

### 2.4 The Effect of FU on Anti‐Inflammation in LPS‐Treated NPCs

**Figure 4:**
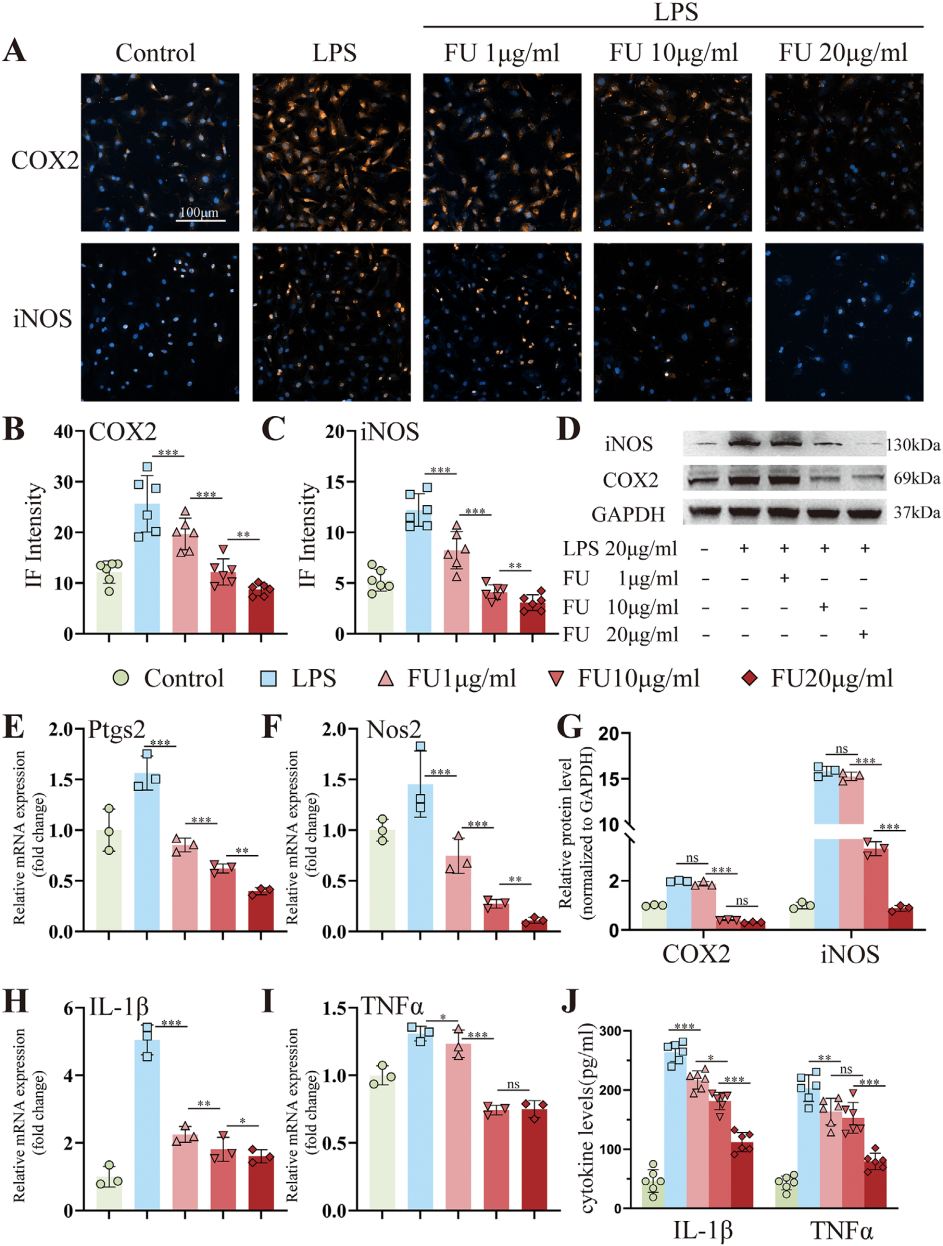
Determine the effective anti‐inflammatory concentration of FU. A) Immunofluorescence of COX2 and iNOS in rat nucleus pulposus cells (NPCs) stimulated by lipopolysaccharide (LPS, 20 µg mL^−1^) and treated with different concentrations of FU for 3 days (scale bar = 100 µm. The blue fluorescence referred to Dapi, while the yellow fluorescence referred to either COX2 or iNOS.). B,C) Semi‐quantitative analysis of the immunofluorescent staining of COX2 and iNOS. D) Western blotting of COX2 and iNOS expression in rat NPCs stimulated by LPS (20 µg mL^−1^) and treated with different concentrations of FU for 3 days. E,F,H,I) Relative mRNA expression of Ptgs2, Nos2, IL‐1β and TNFα. G) Semi‐quantitative analysis of COX2 and iNOS protein expression (n = 3). J) ELISA results for IL‐1β and TNFα concentration (n = 6). All data were presented as mean ± standard deviation, *p < 0.05; **p < 0.01; ***p < 0.001; ns, not significant (one‐way or two‐way ANOVA and Tukey's test compared with each group).

To investigate whether FU exhibited a potential protective effect in degenerated NPCs, an LPS‐treated NPCs inflammatory model was utilized. As shown in Figure S2, Supporting Information, exposure of NPCs to 20 µg mL^−1^ LPS for 24 h could effectively induce inflammation. After 48 h, the expression of inflammatory markers (COX2, iNOS) was reduced with FU treatment in a dose‐dependent manner, respectively, analyzed by WB and IF (**Figure** **4**A–D,G). In addition, RT‐qPCR results revealed that FU diminished the elevated expression of Ptgs2, Nos2, TNFα and IL‐1β induced by LPS treatment (Figure 4E,F,H,I). Meanwhile, the ELISA results were consistent with RT‐qPCR (Figure 4J). To verify whether the concentrations of FU would impact cell viability, a Cell Counting Kit (CCK)‐8 assay was performed. As shown in Figure S3, Supporting Information, the treatments of FU at 1, 10, and 20 µg mL^−1^ had no obvious effect on cell viability. Accordingly, the concentration of FU at 20 µg mL^−1^ could be considered an effective and safe dose for reducing the expression of inflammation. These results demonstrated that FU had excellent anti‐inflammatory effects on NPCs within the inflammatory microenvironment.

### 2.5 Evaluation of Controlled Release and Biocompatibility of GF Microspheres

**Figure 5:**
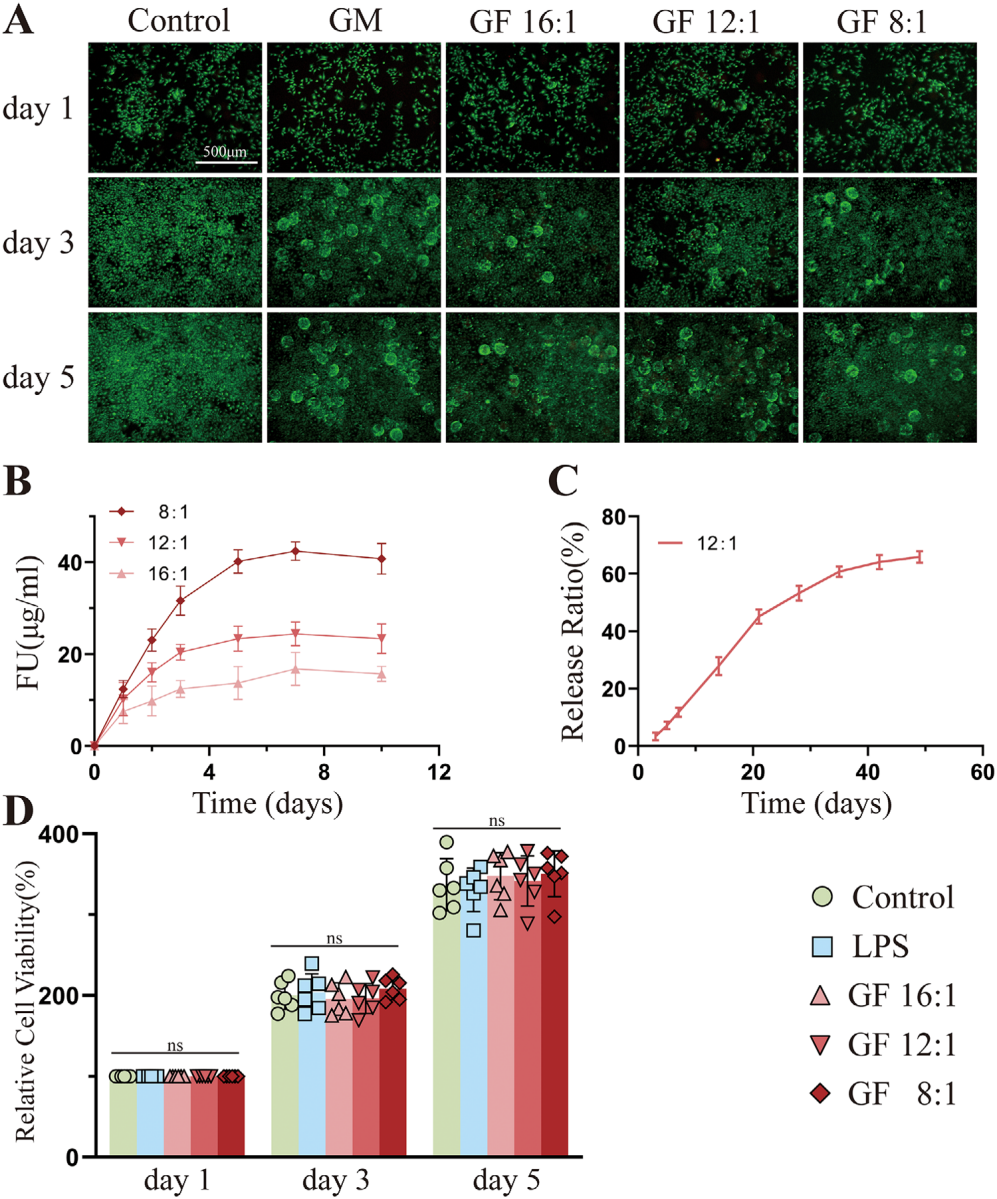
Determine the appropriate ratio of FU in GF microspheres. A) GF microspheres (mass ratio of GelMA and FU, 16:1, 12:1, 8:1) were co‐cultured with LPS‐treated rat NPCs for 1, 3, 5 days and subjected to live‐dead staining (scale bar = 500 µm). B) Release concentration curves of FU from different proportions of GF microspheres at various time points. C) Cumulative release ratio curve of GF microspheres (12:1). D) CCK‐8 assay results on day 1, 3 and 5 of co‐culture of NPCs with different proportions of GF microspheres. All data were presented as mean ± standard deviation, *p < 0.05; **p < 0.01; ***p < 0.001; ns, not significant (one‐way or two‐way ANOVA and Tukey's test compared with each group).

To achieve sustained and effective delivery of FU, GF microspheres were constructed with different mass ratios of GelMA and FU (16:1, 12:1, 8:1) for controlled release. Toluidine blue was employed to assess the release of FU by detecting its sulfate groups. As shown in **Figure** **5**C, the sustained release of FU from GF microspheres was observed for more than 30 days. Under cultivation conditions, GF microspheres across several ratios exhibited similar release rates. Additionally, during the initial 3 days, detection revealed that the 12:1 mass ratio group released FU concentrations closest to 20 µg mL^−1^ (Figure 5B), which was consistent with the effective anti‐inflammatory concentrations of FU validated in previous experiments (Figure 4), indicated that the 12:1 mass ratio group might be suitable for the following experiments. Consequently, a live/dead cell assay was performed (Figure 5A). On day 3, NPCs were well distributed on GM and GF (12:1 mass ratio group) microspheres. By day 5, compared to the control and GM microspheres group, the number of viable cells on the GF microspheres remained high, with no significant increase in the number of dead cells, indicating that the GF microspheres were suitable as scaffolds for NPCs growth and exhibited satisfactory biocompatibility. CCK8 assay additionally revealed that the viability and growth of NPCs in GF microspheres were not obviously impacted, compared to the control and GM groups (Figure 5D). These results confirmed that GF microspheres had functional controlled release property and satisfactory biocompatibility. Next, we explored whether GF microspheres could maintain good anti‐inflammatory properties consistent with FU.

### 2.6 Evaluation of Inflammation Modulation by GF Microspheres

**Figure 6:**
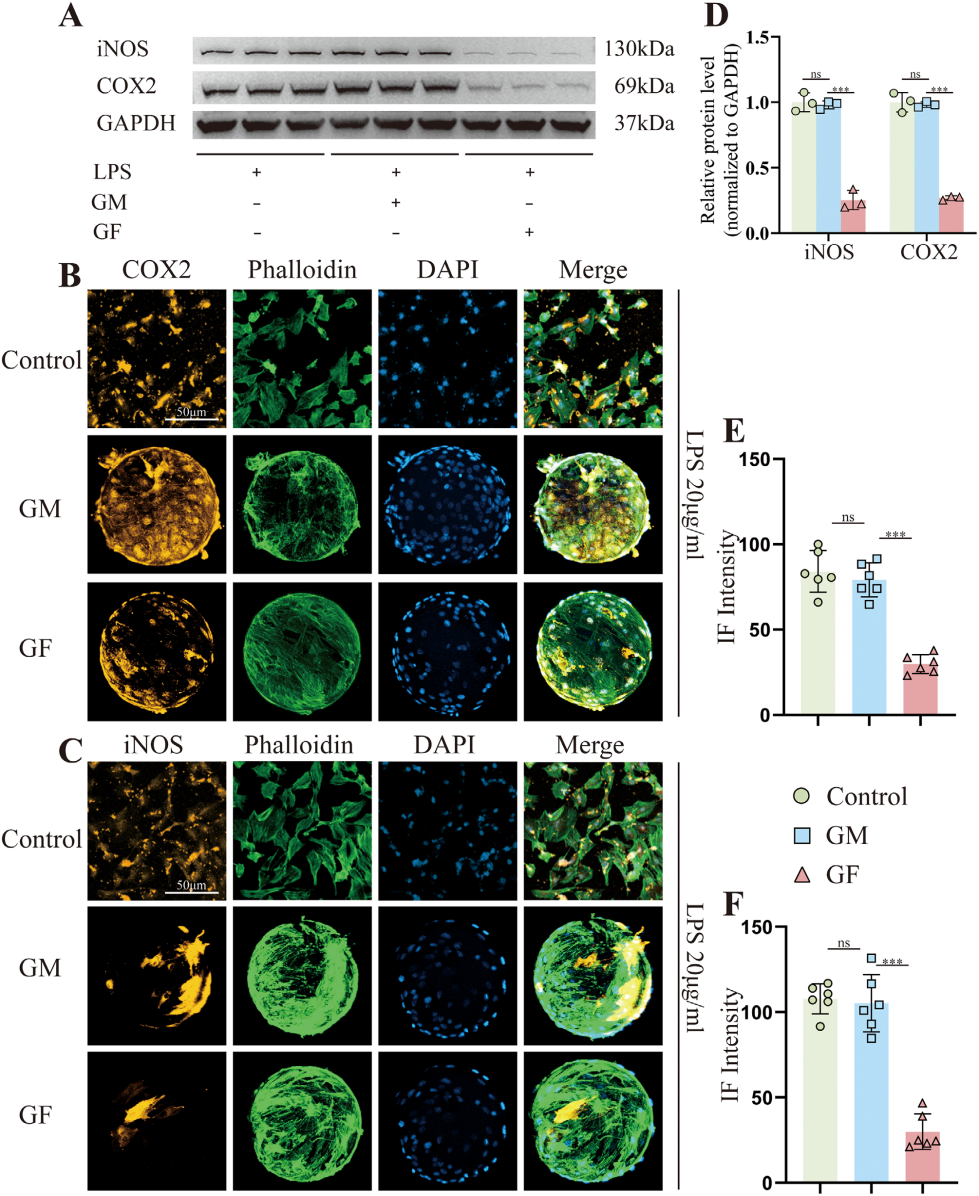
Evaluation of inflammation modulation on LPS‐treated rat NPCs by GF microspheres. A) Western blotting of COX2 and iNOS expression in LPS‐treated rat NPCs co‐cultured with microspheres for 3 days. B) and C) Immunofluorescence of COX2 and iNOS in LPS‐treated rat NPCs co‐cultured with microspheres for 3 days (scale bar = 50 µm). D) Semi‐quantitative analysis of COX2 and iNOS protein expression (n = 3). E) and F) Semi‐quantitative analysis of the immunofluorescent staining of COX2 and iNOS (n = 6). All data were presented as mean ± standard deviation, *p < 0.05; **p < 0.01; ***p < 0.001; ns, not significant (one‐way or two‐way ANOVA and Tukey's test compared with each group).

To assess the regulatory effect of GF microspheres on inflammation, GF microspheres were co‐cultured with LPS‐treated NPCs. RT‐qPCR analysis revealed that following the administration of GF microspheres to LPS‐treated NPCs, the expression of Ptgs2 and Nos2 were significantly downregulated, along with reductions in IL‐1β and TNFα expression (Figure S4A–D, Supporting Information). The ELISA results were consistent with the trend observed in RT‐qPCR, manifested by the reduced expression levels of TNFα and IL‐1β (Figure S4E, Supporting Information). WB results also confirmed the inflammation modulation by GF microspheres, evident in the decreased expression levels of COX2 and iNOS (**Figure** **6**A,D). As shown in Figure 6B,C, the immunofluorescent staining of COX2 and iNOS revealed that the GF microspheres group significantly decreased the fluorescence intensity of these two inflammatory markers, compared to the Control and GM microspheres group (Figure 6E,F). Consequently, GF microspheres were proved to be a delivery vehicle with excellent anti‐inflammatory property, and next would further validate the transfection and ECM repair functions of BLNP to ensure the effectiveness of the BLNP@GF composite microspheres.

### 2.7 The Effect of BLNP on ECM Synthesis in LPS‐Treated NPCs

**Figure 7:**
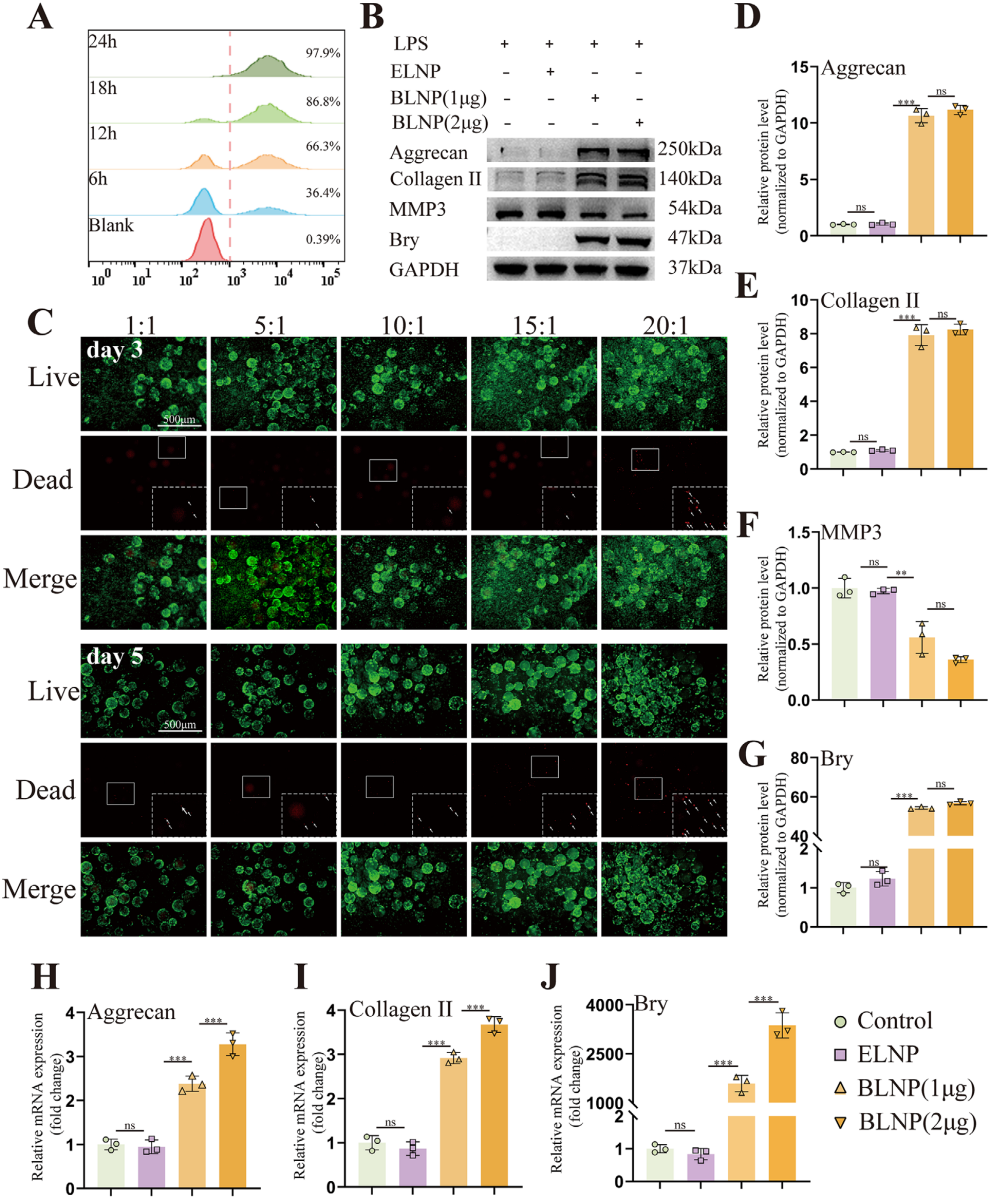
LNP transfection efficiency and BLNP loading ratio. A) Rat NPCs were transfected with LNP containing 2 µg of eGFP mRNA for different times (6, 12, 18, and 24 h), and positive cells with green fluorescence were detected by flow cytometry (n = 3). B) Western blotting of aggrecan, collagen II, MMP3 and Bry protein level in LPS‐treated NPCs transfected with ELNP, BLNP (1 µg of bry mRNA) and BLNP (2 µg of bry mRNA) for 3 days (n = 3). C) Live‐dead staining of rat NPCs cocultured with BLNP@GF microspheres (volume ratio of BLNP and GF, 1:1, 5:1, 10:1, 15:1, 20:1) on 3 and 5 days (n = 3, scale bar = 500 µm. In the white dotted box referred a magnified image of the area of the solid white box, and the white arrows indicated dead cells). D), E), F) and G) Semi‐quantitative analysis of aggrecan, collagen II, MMP3 and Bry protein level (n = 3). H), I) and J) Relative mRNA expression of aggrecan, collagen II and Bry. All data were presented as mean ± standard deviation, *p < 0.05; **p < 0.01; ***p < 0.001; ns, not significant (one‐way or two‐way ANOVA and Tukey's test compared with each group).

To assess the delivery efficiency of LNP, flow cytometry was employed. Flow cytometry analysis revealed that eGFP expression reached 97.9% at 24 h post‐transfection with 2 µg of eGFP mRNA (**Figure** **7**A). To investigate whether BLNP promoted ECM synthesis in NP degeneration, an LPS‐treated NPCs degeneration model was utilized as previously mentioned method. 24 h post‐transfection, RT‐qPCR results showed a significant upregulation of Bry mRNA expression, indicating successful delivery of Bry mRNA by BLNP (Figure 7J). Notably, in addition to Bry mRNA, the expression levels of aggrecan and collagen II mRNA, were also correspondingly increased (Figure 7H,I). WB results showed that BLNP treatment led to upregulation of Bry, aggrecan, and collagen II, and a decrease in MMP3 expression at protein level (Figure 7B,D–G). These results indicated that BLNP effectively delivered Bry mRNA and promoted the upregulation of ECM synthesis in LPS‐treated NPCs. Since then, the biosafety and functionality of the BLNP@GF composite microspheres would be further validated.

### 2.8 Evaluation of Biocompatibility and Regulation of ECM Synthesis by BLNP@GF Microspheres

**Figure 8:**
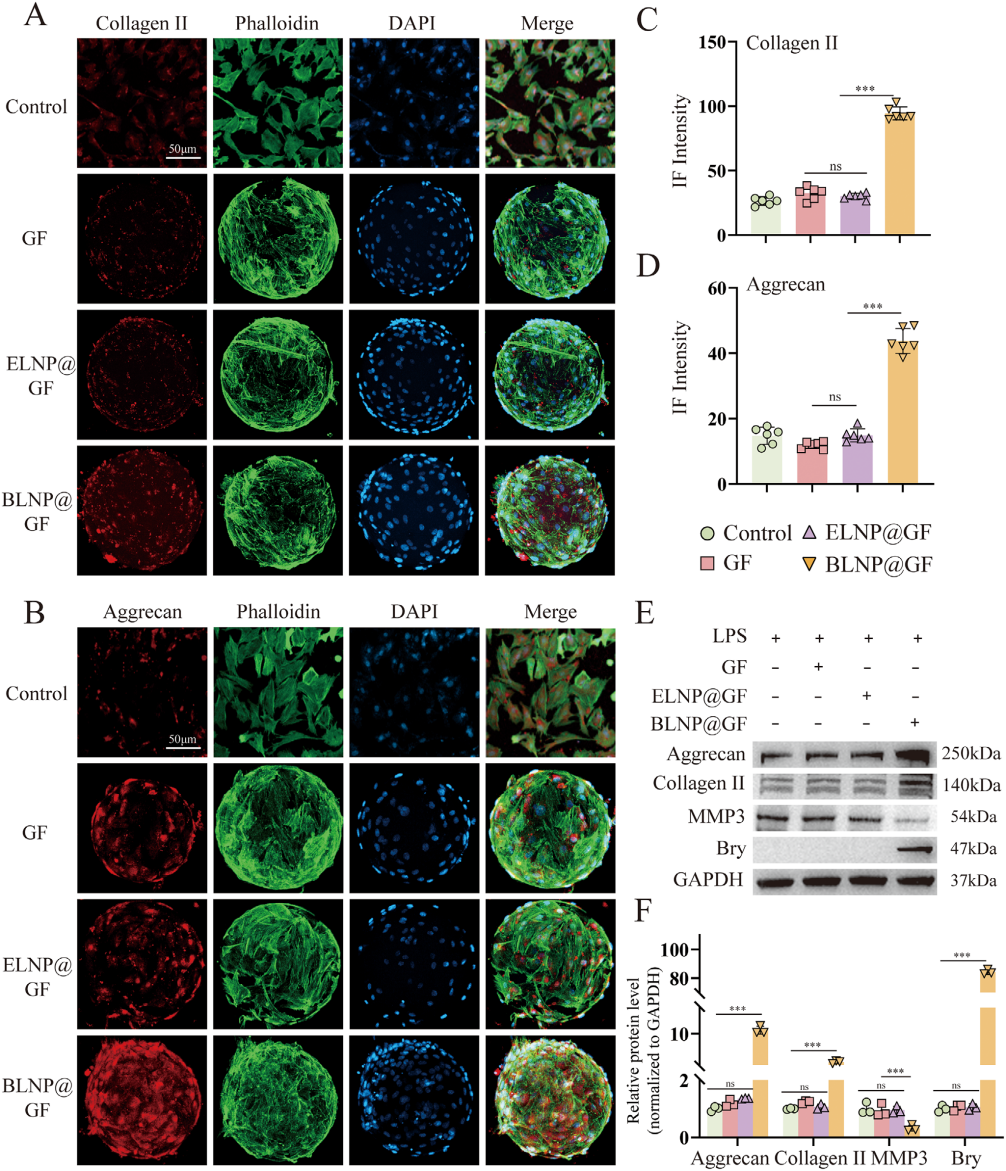
BLNP@GF microspheres upregulated ECM synthesis. A,B) Immunofluorescence of collagen II and aggrecan in LPS (20 ug mL^−1^) ‐treated rat NPCs co‐cultured with different microsphere groups for 3 days (scale bar = 50 µm). C,D) Semi‐quantitative analysis of the immunofluorescent staining of collagen II and aggrecan (n = 6). E) Western blotting of aggrecan, collagen II, MMP3 and Bry expression in LPS‐treated rat NPCs co‐cultured with different microsphere groups for 3 days. F) Semi‐quantitative analysis of aggrecan, collagen II, MMP3 and Bry protein expression (n = 3). All data were presented as mean ± standard deviation, *p < 0.05; **p < 0.01; ***p < 0.001; ns, not significant (one‐way or two‐way ANOVA and Tukey's test compared with each group).

To evaluate the biocompatibility of BLNP@GF microspheres at its appropriate ratio, a live/dead cell assay was performed. As shown in Figure 7C, the volume ratio 15:1 group showed fewer dead cells than the volume ratio 20:1 group on day 3 and day 5, respectively. The CCK8 results were consistent with the live‐dead staining results (Figure S5, Supporting Information). These results indicated that the volume ratio 15:1 group exhibited satisfactory biocompatibility for the following experiments. To evaluate the effectiveness of BLNP@GF microspheres in enhancing ECM synthesis within degenerated NPCs, the composite microspheres were co‐cultured with LPS‐treated NPCs. The IF results revealed a notable increase in the fluorescence intensity of aggrecan and collagen II in the BLNP@GF group (**Figure** **8**A–D). WB results showed that in the BLNP@GF group, the upregulated expression of Bry was accompanied by increased expression of aggrecan and collagen II, as well as decreased expression of MMP3 at protein level. (Figure 8E,F). These results indicated that BLNP@GF microspheres exhibited satisfactory biocompatibility and excellent capacity for enhancing ECM synthesis.

### 2.9 RNA‐Seq Analysis of BLNP@GF‐Induced ECM Remodeling

**Figure 9:**
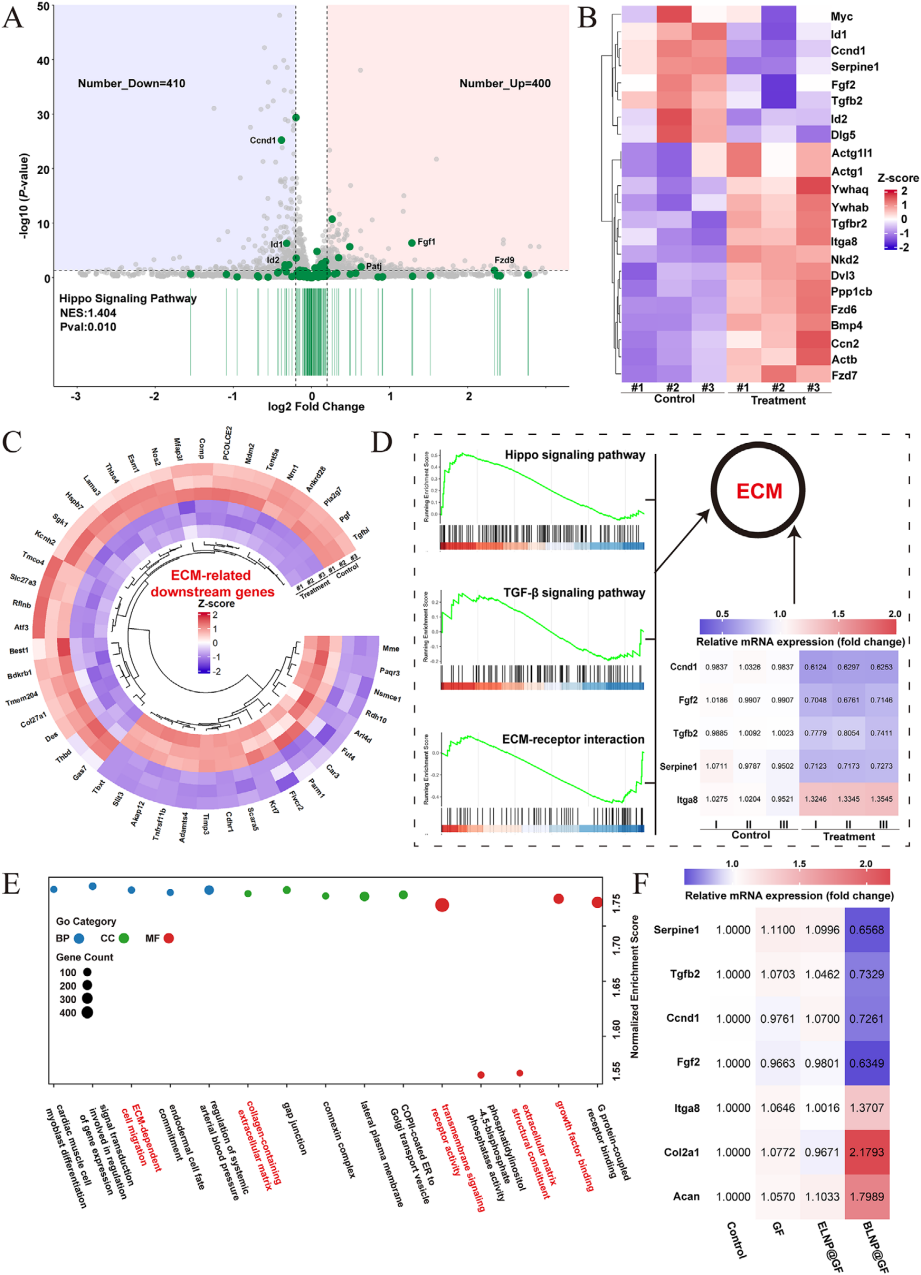
Potential mechanism of ECM synthesis in NPCs treated by BLNP@GF. A) The hybrid volcano plot based on GSEA‐KEGG enrichment analysis of Hippo signaling pathway. B) Heatmap of significantly differentially expressed genes expressed in Hippo signaling pathway and its related signaling pathways. C) Circular heatmap of ECM‐related downstream genes. D) GSEA of Hippo signaling pathway, TGF‐β signaling pathway and ECM‐receptor interaction; Heatmap visualization of RT‐qPCR results for related gene mRNA levels. (All data were presented as mean) E) GSEA‐GO enrichment analysis of biological processes (BP), cellular components (CC) and molecular functions (MF). F) Heatmap visualization of RT‐qPCR analysis of mRNA levels in the degenerated NPCs treated by different groups. (All data were presented as mean).

To systematically explore the biological mechanisms of BLNP@GF in ECM remodeling, we performed RNA Sequencing. First, differential expression analysis of transcriptome data was conducted using a threshold of p.adjust < 0.05 and |log₂FoldChange| > 0.2. A total of 810 significantly differentially expressed genes were screened out, including 400 upregulated genes and 410 downregulated genes. The hybrid volcano plot drawn based on GSEA‐KEGG enrichment analysis showed that the Hippo signaling pathway is one of the most significantly enriched pathways (NES = 1.404, P.adjust = 0.010) (**Figure** **9**A). Multiple core genes in this pathway, such as Ccnd1 and Id1, were downregulated in the treatment group, suggesting that the inhibition of the Hippo pathway may play a crucial role in the transcriptional regulation of ECM‐related genes. In addition, to clarify the expression responses of genes within the Hippo signaling pathway and related signaling pathways, a heatmap of significantly differentially expressed genes was plotted (Figure 9B). Key effector genes such as Id1, Ccnd1, Fgf2, Tgfb2 and Serpine1 were significantly downregulated in the treatment group, while genes such as Itga8 and Bmp4 were upregulated, indicating that their downstream transcriptional activity may maintain the activity of the ECM signaling pathway through feedback mechanisms. Furthermore, we screened out multiple KEGG pathways closely related to ECM but not belonging to the Hippo signaling pathway, extracted representative genes with significant changes, and drew a circular heatmap, revealing the core gene network of ECM remodeling (Figure 9C). Combined with the results of RT‐qPCR experiments, key genes in the Hippo signaling pathway, TGF‐β signaling pathway and ECM‐receptor interaction were selected for verification, confirming that the inhibition of the Hippo signaling pathway and the responses of TGF‐β signaling pathway and ECM‐receptor interaction play crucial roles in the remodeling of the ECM microenvironment (Figure 9D). Then, to comprehensively evaluate the activity changes of ECM‐related pathways, GSEA‐GO enrichment analysis was performed. The top 5 enriched pathways were screened out from biological processes (BP), cellular components (CC) and molecular functions (MF), respectively (Figure 9E). Multiple ECM pathways were significantly enriched in each dimension, specifically including: ECM‐dependent cell migration, collagen‐containing extracellular matrix, extracellular matrix structural constituent, growth factor binding and transmembrane signaling receptor activity. Subsequently, different groups were used to treat degenerated NPCs and the expression changes of related genes were verified by RT‐qPCR (Figure 9F). The GF group and ELNP@GF group had no significant regulatory effects on genes related to the Hippo signaling pathway, TGF‐β signaling pathway and ECM‐receptor interaction, demonstrating that ECM remodeling was a key transcriptional regulatory event under the treatment of BLNP@GF. The increased expression of Col2a1 and Acan also proved that BLNP@GF played a key role in the remodeling of the ECM microenvironment. ECM not only functioned as a structural scaffold in remodeling but also served as a signal transduction platform, a migration guiding module, and a factor storage system, exerting multiple biological functions.

### 2.10 In Vivo Efficacy of BLNP@GF Microspheres in IVDD Treatment

**Figure 10:**
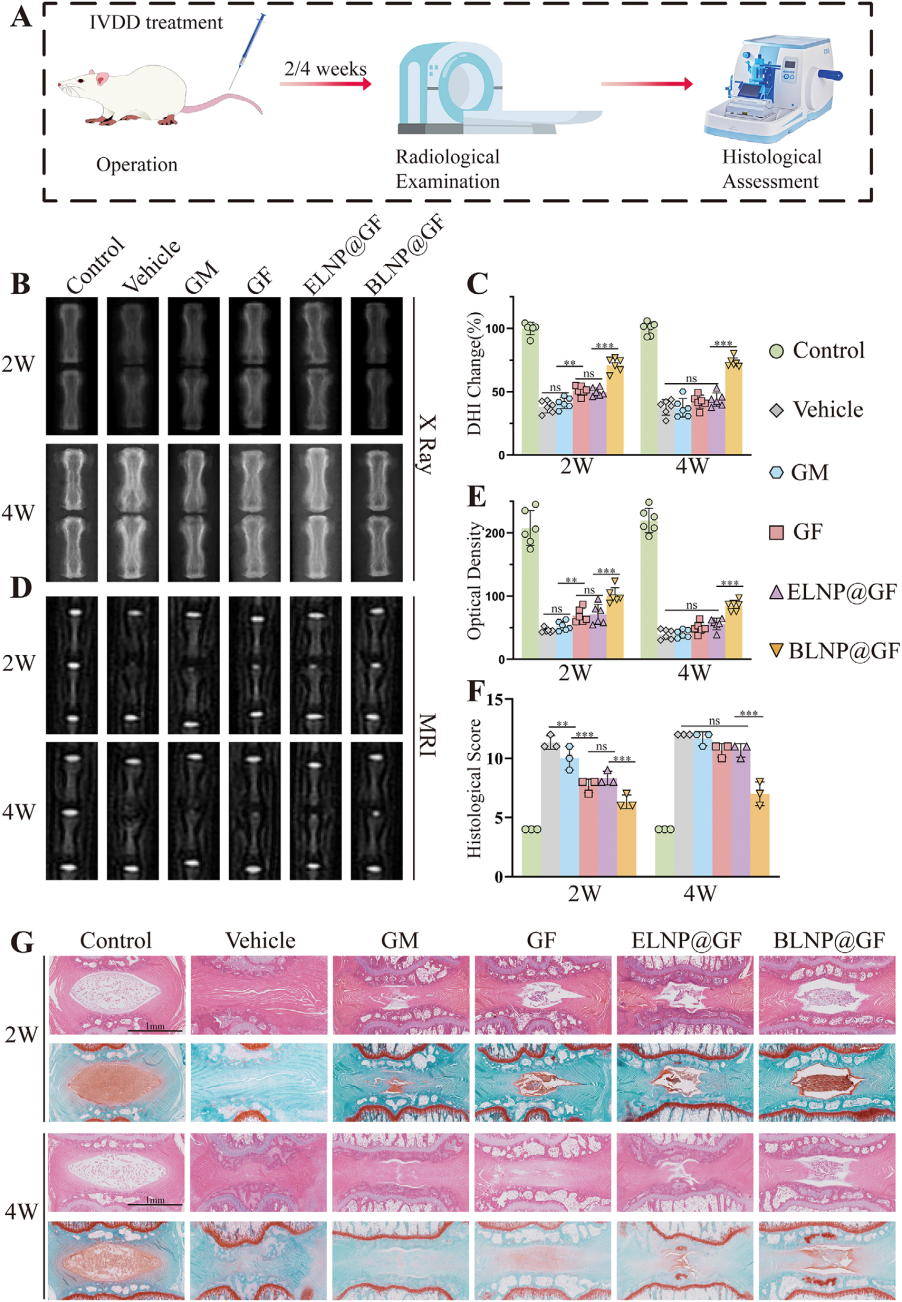
Radiographic evaluation and histological staining of the therapeutic effect of the BLNP@GF microspheres in vivo. A) Diagrammatic illustration of in vivo research design. B,C) X‐ray images and changes in disc height index (DHI) at 2 and 4 weeks. (n = 6). D,E) MRI scans and optical density analysis at 2 and 4 weeks (n = 6). F) and G) H&E and Safranin O‐Fast Green staining and histological score at 2 and 4 weeks (n = 3, scale bar = 1 mm). All data were presented as mean ± standard deviation, *p < 0.05; **p < 0.01; ***p < 0.001; ns, not significant (two‐way ANOVA compared with each group).

A series of deleterious cyclical events, including mechanical overload, inflammatory response, extracellular matrix degradation and cellular depletion, interact to make IVDD a complex and challenging condition to reverse. The ultimate goal of this study was to ameliorate the inflammatory environment within IVD and to enhance the reconstitution of ECM in NPCs in vivo. To investigate the in vivo ability of BLNP@GF microspheres to delay IVDD, a rat model was constructed using needle acupuncture (Figure S6, Supporting Information). The images were performed using radiography and MRI at 2 and 4 weeks post‐treatment (Figure 10A). At 2 weeks postoperatively, the GF and ELNP@GF groups exhibited an increase of 31% and 28% in DHI, respectively, compared to the vehicle group. However, there was no significant difference at 4 weeks. For the BLNP@GF group, DHI at 2 and 4 weeks were obviously increased compared to the other groups (**Figure** **10**B,C). This indicated that the GF group exerted its effect in alleviating inflammation at an early stage, on top of which the delivery of Bry mRNA further enhanced ECM synthesis, thereby restoring the height of the IVD to a certain extent. On T2‐weighted MRI, a bright (white) NP indicates high water content and healthy disc status. Proteoglycan loss decreased water content as degeneration progresses, causing a darker (lower signal) appearance, reflecting disc degeneration. At 2 weeks after surgery, IVD optical density in the GF and ELNP@GF groups increased by 51% and 55%, respectively, compared to the vehicle group. However, no significant difference in IVD optical density was observed at 4 weeks. In addition, the BLNP@GF group exhibited a 1.2‐fold increase and a 1.1‐fold increase compared to the vehicle group (Figure 10D,E). This further suggested that BLNP@GF microspheres enhanced ECM synthesis to retain water content and delay the process of IVDD.

**Figure 11:**
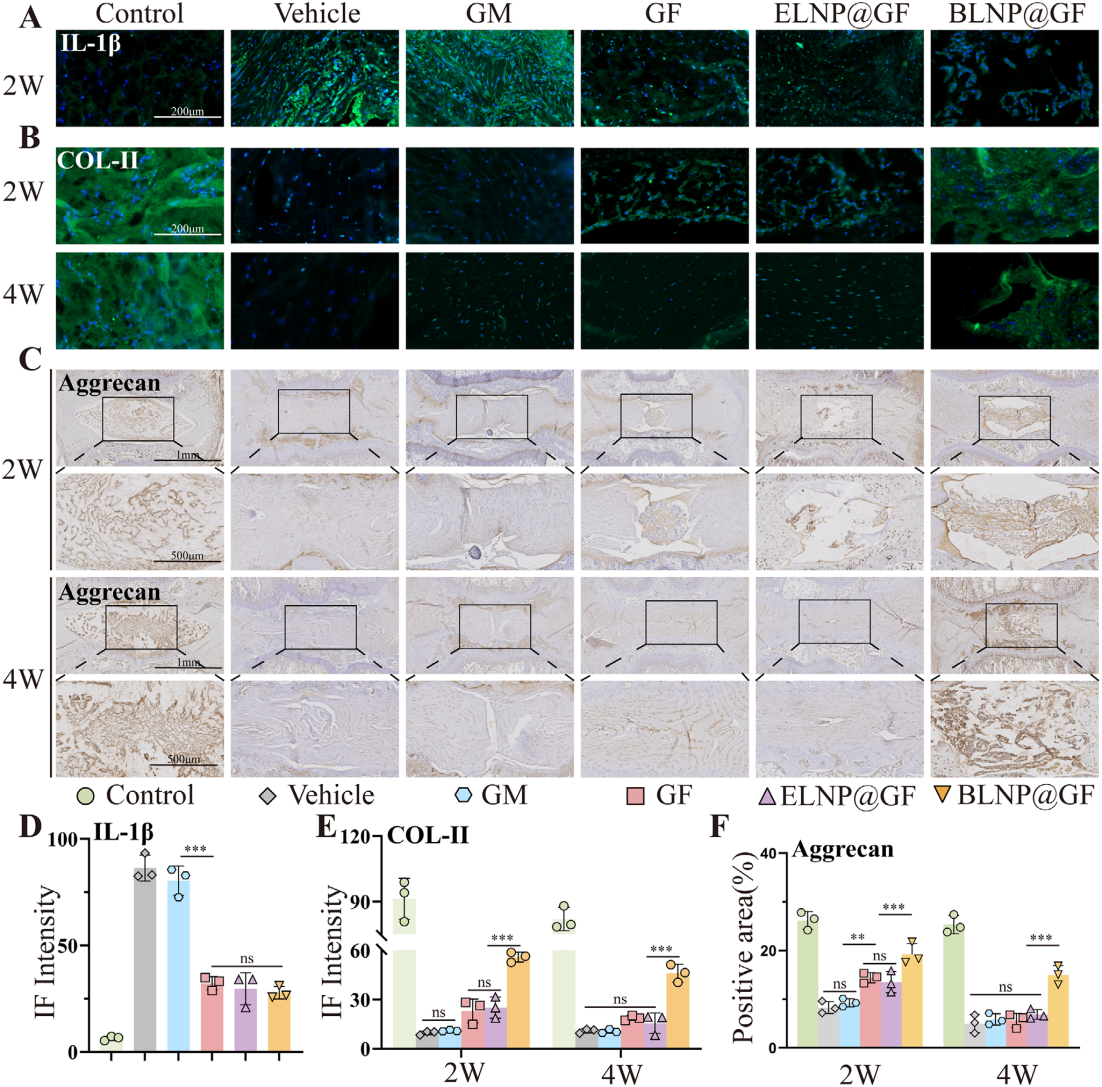
IF and IHC assessment in vivo. A) Immunofluorescence staining of IL‐1β in rat NP tissues at 2 weeks (n = 3, scale bar = 200 µm). B) Immunofluorescence staining of collagen II in rat NP tissues at 2 and 4 weeks (n = 3, scale bar = 200 µm). C) Immunohistochemical staining of aggrecan in rat NP tissues at 2 and 4 weeks (n = 3, scale bar = 1 mm, 500 µm). D) Semi‐quantitative analysis of IL‐1β immunofluorescence (n = 3). E) Semi‐quantitative analysis of collagen II immunofluorescence (n = 3). F) Semi‐quantitative analysis of aggrecan immunohistochemical staining (n = 3). All data were presented as mean ± standard deviation, *p < 0.05; **p < 0.01; ***p < 0.001; ns, not significant (one‐way or two‐way ANOVA and Tukey's test compared with each group).

H&E and Safranin O/Fast Green staining showed that the NP of the control group presented as large and intact elliptical shapes, whereas the NP in the vehicle group was atrophied and almost unobservable at 2 and 4 weeks postoperatively. There was no significant improvement in the GM group compared to the vehicle group. Conversely, there was an increase in NP cellularity and proteoglycan content in the GF and ELNP@GF groups at 2 weeks postoperatively, but no significant difference at 4 weeks. In addition, the BLNP@GF group revealed higher NP cellularity and proteoglycan content at both 2 and 4 weeks (Figure 10F). Histological scores in the BLNP@GF group decreased by 44% at 2 weeks and by 41% at 4 weeks post‐operation, compared with the vehicle group (Figure 10F). Immunohistochemistry (IHC) results demonstrated that the BLNP@GF group exhibited a highly increased expression of aggrecan after 2 and 4 weeks (**Figure** **11**C,F). Similarly, IF was consistent with IHC. BLNP@GF group exhibited significantly enhanced intensity of collagen II at 2 weeks (4.4‐fold higher than Vehicle) and 4 weeks (2.9‐fold higher than Vehicle) (Figure 11B,E). These findings indicated that the sustained and slow release of BLNPs from the material triggered ECM remodeling. Additionally, the significantly decreased intensity of IL‐1β was observed in the GF, ELNP@GF and BLNP@GF groups at 2 weeks (Figure 11A,D). In summary, the in vivo findings showed that the DHI and MRI signals in the BLNP@GF group were significantly superior to those in the Vehicle, GM, GF, and ELNP@GF groups. Histological staining revealed that the BLNP@GF group exhibited significantly higher residual NPC cellularity and increased proteoglycan component deposits. The decreased expression of IL‐1β and the enhanced expression of collagen II and aggrecan suggested that BLNP@GF microspheres could effectively improve the inflammatory microenvironment and modulate ECM synthesis, thereby decelerating the progression of IVDD.

## 3 Discussion

The integrity and health of the IVD are critical to maintaining spinal function, as they are not only support the complex loads of the spine in dynamic motion but also a core element in spinal stability. Throughout the progression of IVDD, the microenvironment undergoes significant alterations characterized by elevated levels of inflammatory molecules, increased oxidative stress, and disruptions in the balance between synthetic and catabolic metabolic processes.^[^ ^34^, ^35^ ^]^ Simultaneously, the local inflammatory milieu facilitates extracellular matrix degradation, thereby exacerbating NPCs degeneration and impeding the reparative process of the ECM.^[^ ^36^ ^]^ Therefore, it should not be overlooked that improving immune imbalances in NPCs and promoting ECM synthesis are pivotal in alleviating IVDD.^[^ ^28^, ^29^ ^]^

The application of biomechanical materials in IVD shows promise in treating IVDD, offering various solutions to modulate the NP microenvironment.^[^ ^37^, ^38^, ^39^ ^]^ Due to the deep location within human tissues and limited blood circulation, injectable biomaterials have long been a focal point of research in IVD repair. Among these, the investigation of injectable microspheres has garnered increasing attention in recent years.^[^ ^29^, ^40^, ^41^ ^]^ Based on this, we have developed a BLNP@GF microsphere delivery method with the dual functionality of alleviating inflammation and promoting ECM synthesis. This platform is suitable for adhesion and growth of the NPCs, addressing both anti‐inflammatory and reparative aspects, thereby providing an effective new approach for the treatment of IVDD.

Proteoglycan (PG) molecules within the NP ECM network exhibit remarkable water‐holding capacity, with high‐density negatively charged PG molecules capable of absorbing approximately three times their own weight in water. This serves as a crucial molecular basis for the excellent mechanical elasticity observed in ECM tissues. Similarly, theoretically speaking, GF microspheres with negative charges should possess similar mechanical properties, very close to the native microstructure of the NP ECM. Fucoidan contains sulfate groups, which confer a negative charge on it. To some extent, this chemical structure bears resemblance to the negatively charged sulfated glycosaminoglycans present in natural ECM tissues. Zhao et al. have also proposed a similar approach in their research, utilizing self‐assembled networks of FU as polysaccharide components to fabricate multi‐component hybrid hydrogels for emulating the natural architecture of cartilage, thus confirming the feasibility of integrating FU‐doped multi‐network hydrogels as scaffolds for tissue repair.^[^ ^42^ ^]^ Excessive addition of FU can lead to repulsion between the negatively charged sulfate groups, thereby adversely impacting the bonding strength and overall properties of the composite material. However, inadequate local application of FU with low concentration may impede its anti‐inflammatory efficacy and lead to insufficient duration for effective anti‐inflammatory treatment. Therefore, we established the effective anti‐inflammatory concentration of FU and determined the loading ratio of FU loaded in GelMA microspheres based on the final concentration derived from FU release experiments. Setting the ratio of 12:1 as the preparation parameters for subsequent experiments ensures both the economic efficiency and effectiveness of GF microspheres. we verified the defensive effect of GF microspheres on the inflammatory microenvironment induced by LPS, which can reduce the expression of inflammatory factors such as COX2, iNOS, IL‐1β, and TNFα in NPCs. Notably, the limited efficacy of the GF group in prolonged in vivo treatment was likely due to the fact that in IVDD, inflammation represents merely one part of the degenerative factors, while a significant enhancement in ECM synthesis was crucial for achieving its biological effects.

Studies on molecular biology‐based therapies have demonstrated considerable potential for delay or reversal of the progression of IVDD. In contrast to the inherent limitations of growth factors, characterized by their short half‐life and rapid degradation within cells, transcription factors exhibit prolonged and stable transfection expression within NPCs, capable of precisely regulating the expression of downstream target genes. Alvarez‐Garcia et al. reported that regulation of autophagy and adaptation to hypoxia by FOXO in primary human NPCs led to enhanced oxidative and inflammatory stress resistance.^[^ ^43^ ^]^ Another study revealed that SOX9 was vital for disc cell survival and phenotypic maintenance, with significant contributions to ECM, cytoskeletal‐related, and metabolic pathways in the NPCs.^[^ ^44^ ^]^ As a key transcription factor involved in mesoderm formation and notochord development, Bry shows promise for biological repair in treating IVDD. Tang et al. reported that sorted Bry‐GFP^+^ cell pellets showed a higher and spatially consistent expression of NP markers and glycosaminoglycans when compared to sorted Bry‐GFP^‐^ cell pellets.^[^ ^45^ ^]^ Our previous studies revealed that Bry exerted a positive remodeling influence on the ECM synthesis in degenerated NPCs and alleviated senescence and apoptosis in NPCs by regulating autophagy.^[^ ^8^, ^9^, ^46^ ^]^ As depicted in Figures 8 and 9, BLNP@GF microsphere, functioning as a delivery platform for BLNP, facilitates its release and transfection, leading to an increased deposition of the ECM upon overexpression of Bry.

The utilization of LNP as an mRNA carrier represents a well‐established mRNA delivery strategy, extensively investigated and applied in the fields of tumor therapy and vaccine development.^[^ ^47^ ^]^ Recently, there has been a growing body of research focused on employing LNPs to deliver mRNA for the treatment of musculoskeletal disorders. For instance, Zheng et al. achieved enhanced intervertebral fusion in osteoporotic rats by encapsulating Usp26 mRNA within matrix metalloproteinase‐responsive GelMA hydrogel microspheres loaded with LNP.^[^ ^48^ ^]^ Sun et al. successfully regenerated local cartilage tissue through localized delivery of LNP‐loaded rhFGF18 mRNA.^[^ ^49^ ^]^ Therefore, in this study, we successfully prepared LNPs that can efficiently load Bry mRNA and characterized the fundamental features of the nanoparticles. The size and potential of nanoparticles play a crucial role in facilitating cellular uptake and enhancing the efficiency of gene transfection. When LNP is administered locally into the NP tissues, it is prone to sudden release or leakage from the needle channel, thereby limiting their potential for sustained therapeutic effects. Therefore, in this study, LNP was chemically grafted onto the surface of GF microspheres, which acted as an effective delivery platform for BLNP while performing anti‐inflammatory functions, helping BLNP to be retained in situ in the focal area and controlling its release rate. Compared to the direct mixing method, grafting LNP onto the surface of GF microspheres ensured efficient delivery and timely transfection of Bry mRNA into NPCs when the cells adhered to hydrogel microspheres. In this study, the delivery of Bry mRNA using BLNP@GF offers a therapeutic strategy for maintaining or restoring Bry expression, thus regulating the complex microenvironment of the IVD.

Nevertheless, our research still has many aspects that can be improved. First, we did not measure the mechanical properties of BLNP@GF microspheres due to their small size and lack of high‐precision instrumentation. Hydrogel surface stiffness has been demonstrated to influence cellular mechanically‐relevant crosstalk between mechanotransduction and metabolism.^[^ ^50^, ^51^, ^52^ ^]^ Next, it must be acknowledged that the specific mechanism of Fu in reducing inflammation in NPCs has not been explored in this study. Research has indicated that the biological functions of FU are closely linked to its source, molecular weight, and sulfate content. Consequently, these factors must be considered as well. Third, the possibility of clinical application of BLNP@GF should be further explored. More long‐term animal experiments, even large animal experiments, are needed to verify the effectiveness of this new strategy.

## 4 Conclusion

In this study, we fabricated GF microspheres and BLNP by microfluidic technology, respectively, and grafted BLNP onto GF microspheres by amide bonding to construct a BLNP@GF hydrogel microsphere. BLNP@GF microspheres had satisfactory biocompatibility, swelling ability and injectability, which could effectively load and stably release BLNP and FU. We demonstrated that FU released from BLNP@GF microspheres was effective in decreasing the expression of inflammatory factors in NPCs. Furthermore, BLNP released from BLNP@GF microspheres was effective in the delivery of Bry mRNA to NPCs, which promoted ECM synthesis. In conclusion, this study presented a valuable, functionalized disc regeneration material, which provided a novel therapeutic strategy for the treatment of IVDD.

## 5 Experimental Section

### Ethics Statement

Ethical approval (IACUC‐2004020) for all surgical interventions, treatments, and postoperative animal care procedures was granted by the Committee of Nanjing Medical University (Nanjing, People's Republic of China). Human NP tissue collection and associated experiments were approved by The Ethics Committee of The Affiliated Suzhou Hospital of Nanjing Medical University, conducted under the guidelines of the Helsinki Declaration (ethical numbers: KL901339).

### Experimental Grouping and Nomenclature

In in vitro experiments, “Control” denoted the control group without treatment. “GM” represented the treatment with GelMA microspheres. “GF” indicated the treatment with GelMA microspheres loaded with FU. “ELNP@GF” signified GF microspheres loaded with empty LNP. “BLNP@GF” denoted GF microspheres loaded with LNP carrying Bry mRNA. In in vivo experiments, “Control” referred to the sham‐operated group without surgery. “Vehicle” represented the untreated group that underwent surgery only. The other groups were named the same as in vitro experiments.

### Reagents and Antibodies

LPS was sourced from Sigma‐Aldrich (L2880, Missouri, USA). FU was purchased from Sigma‐Aldrich (F5631, Missouri, USA). Toluidine blue was purchased from Sigma‐Aldrich (T3260, Missouri, USA). D‐Lin‐MC3‐DMA, cholesterol and DSPC were purchased from MedChemExpress (New Jersey, USA). DMG‐PEG2000‐NH2 was ordered from Shanghai Ponsure Biotech (Shanghai, China). GelMA hydrogel (EFL‐GM‐90) and I2595 photoinitiator were obtained from EFL (Suzhou, China). Additional information regarding the antibodies used in this study can be found in Table S1, Supporting Information.

### Histological and Immunofluorescence Analyses of Human NP Tissues

NP tissues of Pfirrmann grade II (n = 3) were collected from patients diagnosed with lumbar burst fractures and acute lumbar disc protrusion. In contrast, NP tissues with Pfirrmann grade IV (n = 3) were obtained from operated patients with disk degenerative disease. The collected human NP tissues were preserved in 10% formalin for 48 h and subsequently embedded in paraffin wax. The samples were sectioned into 5 µm slices, which were then stained with hematoxylin and eosin (H&E) and safranin‐O/fast green for histological evaluation. The expression level of Bry in human NP tissues was evaluated by co‐staining with carbonic anhydrase‐12 (CA‐12), a marker for NPCs. After dewaxing, gradient hydration, and antigen retrieval, the sections were blocked in 1% bovine serum albumin (BSA) and incubated with primary antibodies (Bry and CA‐12, (Table S1, Supporting Information)) overnight at 4 °C. After rinsing three times, the sections were labeled with the appropriated Alexa Fluor conjugated secondary antibodies and counterstained with DAPI (Beyotime, Shanghai, China). The immunofluorescence images were obtained using a Zeiss laser confocal microscope (LSM800; Carl Zeiss, Oberkochen, Germany).

### Synthesis of Materials—Preparation of GM and GF Microspheres

GM hydrogel microspheres were synthesized utilizing a water‐in‐oil microfluidic technique. The microfluidic extrusion equipment, characterized by an outer needle diameter of 30 G and an inner one of 21 G, was linked to a microfluidic propulsion system through a silicone conduit. The outer needle functioned as the continuous phase for the co‐flow shearing process and was connected to a solution of liquid paraffin containing 5% (w/w) span 80. The inner needle facilitated the dispersed phase, which consisted of an 8% (w/v) GelMA solution in water and a 0.5% (w/v) I2595 photoinitiator. By setting the flow rate of the GelMA solution to 1:40 that of the oil phase, uniform spherical droplets were consistently produced at the phase boundary. Once the formation of GM droplets was confirmed to be stable, they were collected into a beaker containing the oil phase and kept on ice. Every 10 to 15 min, the beaker was taken out, and the droplets were fully exposed to UV light (405 nm) while being gently stirred, causing them to solidify as separate microspheres rather than clumping together. The resulting cross‐linked hydrogel microspheres were then subjected to three rounds of washing with acetone and five washes with 75% ethanol, followed by five washes with deionized water, with the deionized water being refreshed every 3 h to eliminate residual photoinitiator and oil. Finally, the purified microspheres were frozen at −80 °C overnight and subsequently freeze‐dried for at least 48 h to yield porous GM microspheres. To fabricate GF microspheres, 8% (w/v) GelMA aqueous solution and 0.5% (w/v) I2595 photoinitiator were mixed with different masses of FU (the mass ratio of GelMA and FU, 16:1, 12:1, 8:1) and used the same water‐in‐oil method as above.

### Synthesis of Materials—Preparation of mRNA

Genetic sequences (EGFP, Bry) in the PVAX1 plasmid were constructed in this study. We commissioned VectorBuilder (Guangzhou, China) to synthesize plasmids. After the BspQI restriction enzyme (NEB, R0712L) treatment, the linearized plasmid was purified with the Monarch PCR & DNA Cleanup Kit (NEB, T1030L). and then transcribed into mRNA with the HiScribe T7 High Yield RNA Synthesis Kit (NEB, E2050L). The production of mRNA in vitro transcription was purified with the Monarch Spin RNA Cleanup Kit (NEB, T2040L). The mRNA concentration was ultimately quantified using a NanoDrop 2000 spectrophotometer (Thermo Scientific, Cambridge, MA, USA).

### Synthesis of Materials—Preparation of BLNP

LNPs/mRNA refers to a type of nanoparticle that includes four lipid components along with mRNA. The formulation (50:38.5:10:1.5) included ionizable lipids (D‐Lin‐MC3‐DMA), cholesterol, DSPC, and DMG‐PEG2000‐NH2, which were homogeneously mixed in the ethanol phase (12 mM), whereas mRNA was kept in a 50 mM citrate buffer at pH 4. Rapid mixing of the aqueous and ethanol phases was achieved using a microfluidic mixing device (LNP‐B1, FluidicLab, Shanghai, China) with a volume ratio of 3:1. The newly formed LNPs/mRNA blend was then dialyzed against phosphate‐buffered saline (PBS) at pH 7.4, using a 30 kDa centrifugal filter (UFC903096, Millipore). The completed LNPs/mRNA preparations were either used immediately or kept at 4 °C for later use.^[^ ^31^ ^]^ To measure the encapsulation efficiency of the LNPs/mRNA complexes, the Ribogreen assay (R11491, Thermo Scientific) was applied. To find the amount of free mRNA within the total mRNA content, the LNPs/mRNA samples were processed with TE buffer for total mRNA and with a 2% TE‐Triton buffer for free mRNA. After incorporating the Ribogreen reagent, a fluorescence microplate reader was used to measure the intensity of fluorescence. The encapsulation efficiency was calculated using the following formula:

(1) $$\mathrm{Encapsulation}\;\mathrm{Efficiency}\left(\%\right)=\left(\mathrm{mRN}A_{\mathrm{total}}-\mathrm{mRN}A_{\mathrm{free}}\right)/\mathrm{mRN}A_{\mathrm{total}}\times 100\%$$

### Synthesis of Materials—Preparation of BLNP@GF Microspheres

To activate GelMA@FU surface carboxyl groups for binding to the amino group on the BLNP, an EDC/NHS two‐step method was used.^[^ ^53^ ^]^ Lyophilized GF microspheres, 16 mg of 1‐(3‐Dimethylaminopropyl)‐3‐ethylcarbodiimide (EDC), and 24 mg of N‐Hydroxy succinimide (NHS) were sequentially introduced into a 2 mL of MES buffer (pH 6.0). Incubated for 30 min at 37 °C. After centrifugation, GF microspheres were resuspended in different volumes of BLNP solution and then incubated at 4 °C for 24 h. Subsequently, they were centrifuged again and rinsed three times with deionized water.

### Characterization of Different Composite Microspheres—Scanning Electron Microscopy

GM and GF microspheres were separately secured with conductive adhesive tape, mounted onto the sample stage of SEM, and then coated with a layer of gold using a sputter coater (Quorum Technologies in Lewes, UK). Once prepared, the samples were introduced into the SEM chamber (Crossbeam 350, Carl Zeiss AG, Oberkochen, Germany). The microscope's accelerating voltage was adjusted to 10 KV to properly examine the structural details and morphology of microspheres.

### Characterization of Different Composite Microspheres—Transmission Electron Microscopy

The LNP solution was applied dropwise to the copper mesh, dispensing 2 µL at a time, and after 5 min, the excess liquid was carefully aspirated. This procedure was repeated three times to ensure effective application. Subsequently, 2 µL of 4% phosphotungstic acid was added dropwise for staining for 20 min. After removing the excess liquid, 2 µL of deionized water was added dropwise to wash away the remaining staining solution, repeating this process three times. The copper mesh was then dried for 24 h. The voltage was set at 120 kV to run TEM (Titan G2 60–300, FEI Company, Hillsboro, OR, USA) to observe the morphology of the LNP.

### Characterization of Different Composite Microspheres—Dil Staining

To evaluate the grafting of BLNP on the surface of GF, we used a Cell Plasma Membrane Staining Kit with DiI (Red Fluorescence) (C1991S, Beyotime, Suzhou, China).^[^ ^53^ ^]^ Dil is a fluorescent probe that binds to phospholipid bilayer membranes. In brief, Dil(400X), stain enhancer(400X) and staining buffer were configured in a ratio of 1:1:398 to form a working solution. BLNP@GF microspheres were resuspended in the working solution and then incubated at 37 °C for 5–20 min in the dark. After rinsing three times, the stained microspheres were transferred to a confocal dish and observed using a confocal laser microscope (Zeiss LSM900; Carl Zeiss, Oberkochen, Germany).

### Characterization of Different Composite Microspheres—Particle Size and Zeta Potential

The size, polydispersity index (PDI) and zeta potential of LNP and BLNP were measured using a Zetasizer (Malvern Zetasizer Nano ZS90, UK) for Dynamic light scattering (DLS) analysis.

### Characterization of Different Composite Microspheres—Fourier‐Transform Infrared Spectroscopy

The formation of the amide bonds between GF and BLNP and the characterization of different functional groups carried by GM and GF were detected by scanning each sample 128 times by an FTIR spectrometer (Thermo Fisher Scientific) with a resolution of 4 cm^−1^ and a range of ≈500–4000 cm^−1^.

### Characterization of Different Composite Microspheres—Swelling Test

GF microspheres were immersed in PBS solution at room temperature to determine the swelling capacity of the sample. The initial mass of the sample was positioned at W_0_ and weighed at different times until the mass at swelling equilibrium was W_S_. Swelling rate (SR) = (W_S_ ‐W_0_)/ W_0_ × 100%.

### Characterization of Different Composite Microspheres—Elemental Analyses

Elemental analyses of the GM and GF microspheres’ surfaces were conducted using Energy Dispersive Spectrometer (EDS, Crossbeam 350, Carl Zeiss AG, Oberkochen, Germany).^[^ ^54^ ^]^

### Detection of FU Release from GF Microspheres

In order to prepare GF microspheres loaded with an appropriate proportion of FU for effective anti‐inflammatory action, we tested the release characteristics of FU from GF microspheres (the mass ratio of GelMA and FU, 16:1, 12:1, 8:1). 200 mg of GF microspheres was immersed in 3 mL of PBS, and shaken at 37 °C. At the predetermined time intervals, 1.5 mL of the solution was removed for analysis and 1.5 mL of fresh PBS was added back for continuing incubation. Then, referring to the method in the previous research,^[^ ^55^, ^56^ ^]^ after the reaction of FU with quantitative toluidine blue, the absorbance of residual toluidine blue was measured by a UV–vis spectrophotometer (TU‐1900, Persee, Beijing, China) at a wavelength of 630 nm to determine the concentration of FU accordingly.

### Biocompatibility of the Composite Microspheres

To assess the biocompatibility of composite microspheres with NPCs, a live/dead staining assay was performed. GF (16:1, 12:1, 8:1) or BLNP@GF (1:1, 5:1, 10:1, 15:1, 20:1) microspheres were placed at the bottom of a 24‐well plate, with ≈200 mg microspheres in each well seeded with NPCs. The co‐culture was terminated on days 1, 3, and 5. After rinsing, the Live/Dead Assay Kit (C2015M, Beyotime, Shanghai, China) was employed and incubated at 37 °C for 30 min. Subsequently, the cell morphology was examined using an inverted fluorescence microscope.

To assess cell viability, GF or BLNP@GF microspheres were co‐cultured with NPCs. On days 1, 3, and 5 post‐co‐culture, a Cell Counting Kit‐8 (CCK‐8; Biosharp, Hefei, China) assay was used. The absorbance of each well was measured using a microplate reader at 450 nm.

### Isolation and Culture of NPCs

Male Sprague Dawley (SD) rats (8–9 weeks old) were euthanized by intraperitoneal injection of an overdose of sodium pentobarbital. Under aseptic conditions, NP tissues were carefully harvested from each coccyx (Co1‐Co5)and subsequently digested using 0.5% type II collagenase at 37 °C for 2 h. The isolated NPCs were cultured in Dulbecco's modified Eagle's medium/nutrient mixture F‐12 (DMEM/ F‐12, Invitrogen, Carlsbad, CA) supplemented with 12% fetal bovine serum (FBS, Invitrogen) and 1% antibiotic (streptomycin and penicillin). NPCs were incubated in an incubator with 5% CO_2_ at 37 °C. The culture medium was replaced every alternate day, and NPCs derived from the second or third passage were used for the subsequent experiment.

### Cell Transfection

According to the previously mentioned methods, mRNA was prepared and encapsulated in LNP. When reaching 40%–50% confluence, NPCs were transduced with 2 µg of mRNA through LNP. After 24 h of the transduction, the culture medium was refreshed. NPCs samples were collected for further analysis at 72 h post‐transfection.

### Flow Cytometry

After transfection of NPCs with 2 µg of eGFP mRNA through LNP, the cells were harvested at several time points to assess transfection efficiency. In brief, the eGFP mRNA delivered by LNP was translated and expressed upon entry into NPCs, which could be detected by flow cytometry (FACSCanto II, BD, New Jersey, USA) due to its emission of green light. The results were analyzed using FlowJo software version 10.8 (BD Life Sciences).

### Western Blot

Following the co‐culture protocol, NPCs were treated with lipopolysaccharide (LPS) at a concentration of 20 µg mL^−1^ and cultured for 3 days. The cells were then harvested and lysed with a radioimmunoprecipitation assay (RIPA) supplemented with 1% phenylmethanesulfonyl fluoride (PMSF). The concentration of the extracted proteins was then determined using a bicinchoninic acid assay (BCA) kit (Beyotime, Shanghai, China). Equivalent amounts of the denatured protein samples were separated by 10% sodium dodecyl sulfate‐polyacrylamide gel electrophoresis (SDS‐PAGE) and subsequently transferred onto polyvinylidene difluoride (PVDF) membranes. Membranes were blocked with 5% skim milk and then incubated with primary antibodies (Table S1, Supporting Information) at 4 °C overnight. After rinsing with Tris Buffered Saline with Tween‐20 (TBST), membranes were incubated with corresponding secondary antibodies at room temperature. Protein levels of bands were visualized by enhanced chemiluminescence (EFL) and analyzed using ImageJ software, which facilitated the quantification of protein expression levels by measuring the gray values of bands.

### Immunofluorescence

NPCs were cultivated alongside different groups, as detailed in the previous description. After being fixed in 4% paraformaldehyde, specimens were permeabilized with 0.3% Triton X‐100 for 15 min and then blocked with 5% bovine serum albumin (BSA) (Sigma‐Aldrich, USA) for 1 h. Subsequently, specimens were incubated with primary antibodies (Table S1, Supporting Information) at 4 °C overnight in order to bind respective antigens. After rinsing, the samples were further incubated with appropriate secondary antibodies for 2 h at room temperature. To visualize the cytoskeleton and nuclei, the samples were stained with phalloidin and DAPI. The IF images were obtained using a confocal laser microscope (Zeiss LSM900; Carl Zeiss, Oberkochen, Germany). A semi‐quantitative analysis of the fluorescence was conducted using ImageJ software.

### Quantitative Real‐Time Polymerase Chain Reaction

Total RNA from NPCs was extracted using TRIzol reagent (Invitrogen, Waltham, MA). According to the manufacturer's instructions, RNA specimens (1 µg) were reverse‐transcribed into cDNAs using a Prime Script Reverse Transcription Kit (Vazyme, Nanjing, China). RT‐qPCR was performed using the ABI 7500 Real‐Time PCR system (Applied Biosystems, Foster City, CA, United States). During the analysis, the relative gene expression of all samples was normalized to the β‐actin gene. All the primer sequences (Tsingke, Beijing, China) used in the study are detailed in Table S2, Supporting Information.

### Enzyme‐Linked Immunosorbent Assays

The medium supernatants of NPCs cultured with different groups were collected. The respective concentrations for IL‐1β and TNFα were measured by ELISA kits (EK301BHS, EK382HS, MultiSciences (LiankeBio), Hangzhou, China) in accordance to the manufacturer's instructions.

### RNA Sequencing and Data Analysis

Total RNA was isolated using TRIzol reagent (Invitrogen, CA, USA). RNA samples from NPCs in the control and treatment groups was first used to clarify differences in gene expression and encoded proteins between them. Additionally, NPCs co‐cultured with both the control and treatment groups were used to explore the mechanisms and pathways of BLNP@GF microspheres. RNA purity and quantification were determined using a NanoDrop 2000 spectrophotometer (Thermo Fisher Scientific, Waltham, MA, USA), and RNA integrity was assessed using an Agilent 2100 Bioanalyzer (Agilent Technologies, Santa Clara, CA, USA). Libraries were then constructed using the VAHTS Universal V6 RNA‐seq Library Prep Kit according to the manufacturer's instructions. Transcriptome sequencing and analysis were performed by OE Biotech Co., Ltd. (Shanghai, China).

### Establishment of the Rat IVDD Model

Seventy‐two male SD rats aged 12 weeks with an average weight of 380 ± 20 g were obtained and randomly divided into two groups of 36 rats each. One group was analyzed 2 weeks after the operation, and the other group was analyzed 4 weeks post‐operation. The rats were housed in a well‐ventilated environment at a consistent temperature of 21 °C and a 12‐h alternating light‐dark cycle. Rats were anesthetized with an intraperitoneal injection of 0.3% pentobarbital. A standardized surgical procedure was performed to establish an IVDD model as previously described.^[^ ^57^ ^]^ In brief, degeneration of the seventh to ninth caudal vertebrae was induced with a 20‐gauge needle puncture. To induce degeneration successfully, the needle was rotated for 5 s and maintained its position for 30 s. Each group of 36 rats was additionally divided randomly into six subgroups: the Control group, Vehicle group, GM group, GF group, ELNP@GF group, and BLNP@GF group, with six rats per subgroup. ≈15 µL of PBS was injected into the Vehicle group and 15 µL of the GM, GF, ELNP@GF and BLNP@GF microspheres were respectively injected into their corresponding groups through a 28‐gauge micropump syringe.

### Radiographic Evaluation of Animal Models

At both 2 and 4 weeks after surgery, rats from each group (n = 6) were examined with X‐ray and MRI prior to euthanasia. The rats were positioned supine with their tails straightened on a mammography device (GE Healthcare, Chicago, IL). Radiographs of the rats were obtained using a collimator to‐film distance of 66 cm, an exposure of 63 mAs, and a penetrating voltage of 35 kV. The X‐ray images were analyzed using ImageJ software to determine the disc height index (DHI) as a percentage. For the MRI scans, sagittal T2‐weighted images were captured using a 1.5‐T system (GE; fill time, 3000 ms; echo time, 80 ms; field of view, 200 mm × 200 mm; scan thickness, 1.4 mm). The level of disc signal intensity was quantitatively assessed using ImageJ software.

### Histological Analysis

After radiographic assessment, rats from each experimental group were euthanized. Subsequently, the rat caudal disks samples were harvested and then fixed by immersion in 4% paraformaldehyde for 48 h. The samples underwent decalcification in a 10% ethylenediaminetetraacetic acid (EDTA) solution for 7 weeks, followed by paraffin embedding. The specimens were then cut into sections with a thickness of ≈5 µm. To assess the changes in tissue structure and collagen content within the IVD, the sections were stained with H&E and safranin‐O/fast green. The histological scores were evaluated in accordance with criteria previously described by Masuda.^[^ ^58^ ^]^

### Immunohistochemical Staining and IF

Following deparaffinization, graded dehydration and antigen retrieval, the paraffin sections were respectively incubated with corresponding primary antibodies (Table S1, Supporting Information) at 4 °C overnight. After rinsing, secondary antibodies were added dropwise for incubation. For IHC assays, the sections were counterstained with hematoxylin finally. The images of each group were captured with a Zeiss Axio Scan.Z1 Digital Slide Scanner (Carl Zeiss AG, Oberkochen, Germany) and quantified using Image J software.

### Statistical Analysis

Each experimental dataset was independently measured at least three times, with results expressed as mean values accompanied by their standard deviations (SD). Statistical analysis and the creation of graphs were facilitated by GraphPad Prism version 8.0 (Boston, Massachusetts, USA). To compare the statistical variance among different groups, this work employed either one‐ or two‐way analysis of variance (ANOVA). Statistical significance was determined at *p <* 0.05, *p <* 0.01, and *p*<0.001.

## Conflict of Interest

The authors declare no conflict of interest.

## Author Contributions

Y.G., W.S., and X.L. contributed equally to this work. Y.G.: Writing – original draft, Methodology, Investigation, Formal analysis, Data curation. W.S.: Resources, Methodology, Investigation, Formal analysis. X.L.: Writing – review & editing, Methodology, Formal analysis. H.Y.: Writing – review & editing, Methodology, Data curation. Y.W.: Supervision, Formal analysis. Y.X.: Investigation, Formal analysis. C.Y.: Formal analysis, Data curation. C.Y.: Formal analysis. C.S.: Software, Methodology. R.P.: Writing – review & editing, Methodology. T.X.: Writing – review & editing, Supervision, Funding acquisition, Formal analysis, Conceptualization. H.P.: Writing – review & editing, Supervision, Methodology. J.S.: Supervision, Methodology, Funding acquisition, Conceptualization.

## Supporting information



Supporting Information

Supporting Information

## Data Availability

The data that support the findings of this study are available on request from the corresponding author. The data are not publicly available due to privacy or ethical restrictions.
