# Supplementary material for: Brachyury‐Activated Fucoidan Hydrogel Microspheres Rejuvenate Degenerative Intervertebral Discs Microenvironment
Source: Adv Sci (Weinh). 2025 Jun 20;12(34):e04195. doi: 10.1002/advs.202504195 (PMC12442695; doi:10.1002/advs.202504195)
Supplement: Supplementary file 1 — Supporting Information [file ADVS-12-e04195-s001.docx]

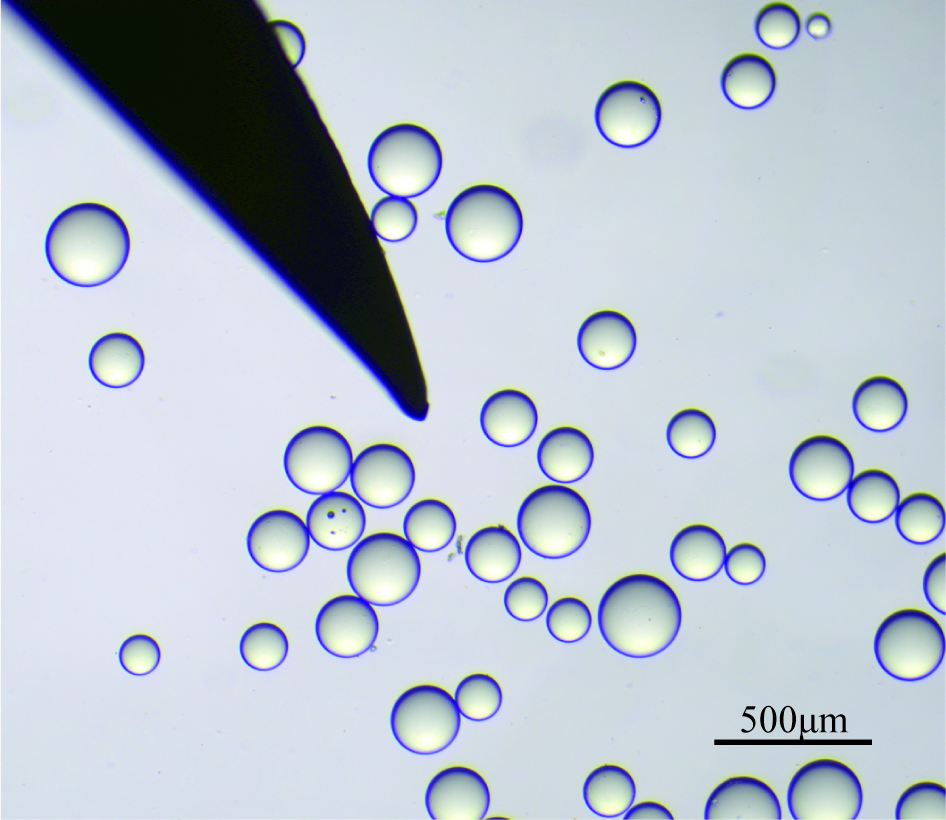


**Supplementary Figure S1.** Validation of BLNP@GF microsphere injection performance though a 28G syringe.


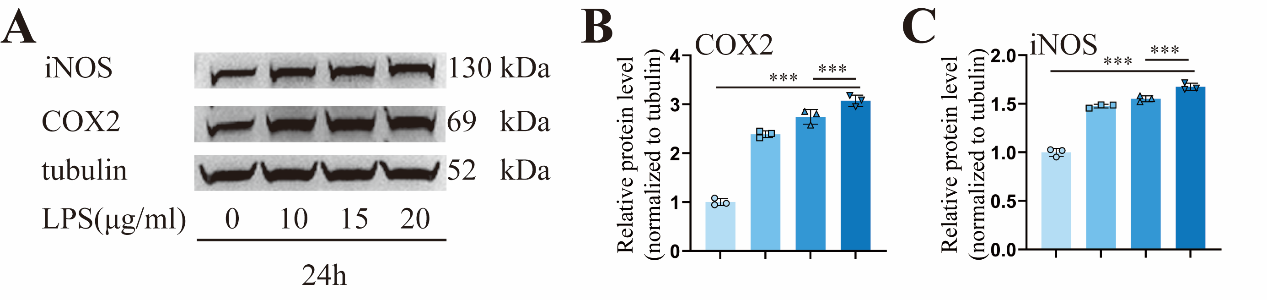


**Supplementary Figure S2.** **Determination of appropriate concentration of LPS-induced inflammation in rat NPCs.** A) Western blotting of COX2 and iNOS expression in rat NPCs stimulated by different concentration of LPS. B) and C) Semi-quantitative analysis of COX2 and iNOS protein expression (n = 3). All data were presented as mean ± standard deviation, **p <* 0.05; ***p <* 0.01; ****p <* 0.001; ns, not significant (one-way ANOVA and Tukey’s test compared with each group).


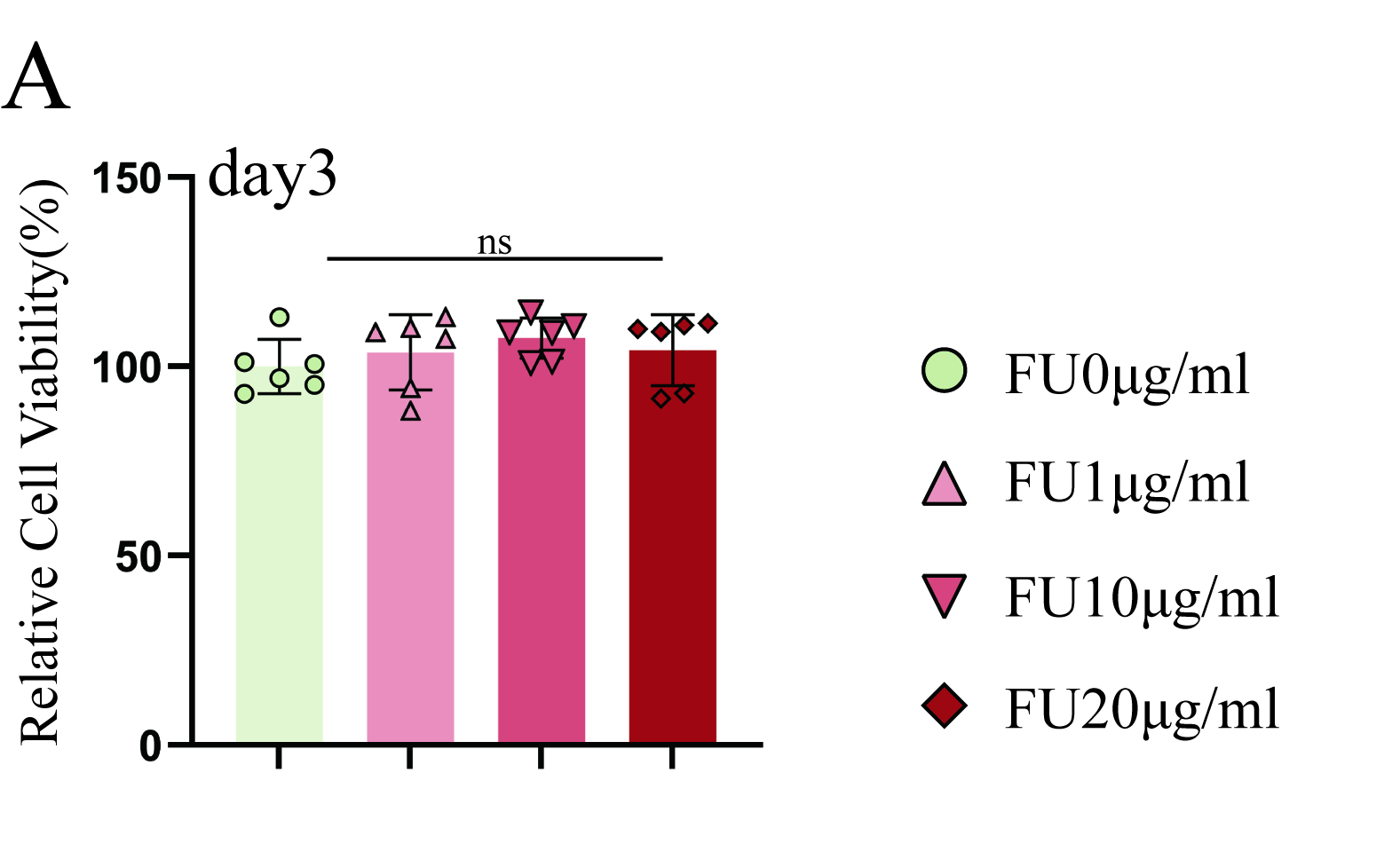


**Supplementary Figure S3.** A) The results of CCK-8 detection of LPS-treated rat NPCs with different concentrations of FU treatment at day 3 (n = 6). All data were presented as mean ± standard deviation, **p <* 0.05; ***p <* 0.01; ****p <* 0.001; ns, not significant (one-way ANOVA and Tukey’s test compared with each group).


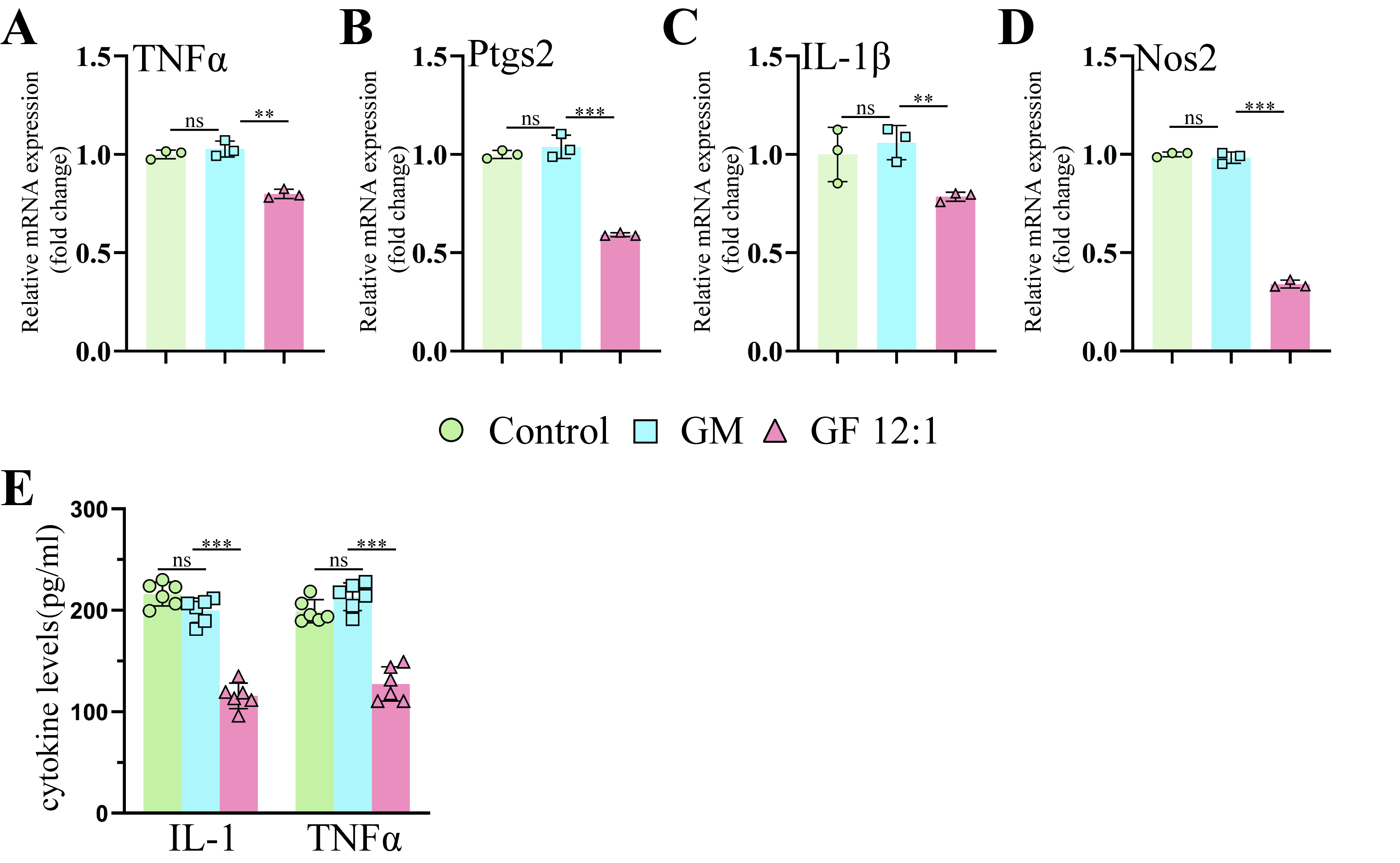


**Supplementary Figure S4.** A), B), C, D) Relative mRNA expression levels of Ptgs2, Nos2, IL-1β and TNFα in LPS-treated rat NPCs co-cultured with microspheres for 3 days (n = 3). E) ELISA results for IL-1β and TNFα concentration in LPS-treated rat NPCs co-cultured with microspheres for 3 days (n = 6). All data were presented as mean ± standard deviation, **p <* 0.05; ***p <* 0.01; ****p <* 0.001; ns, not significant (one-way or two-way ANOVA compared with each group).


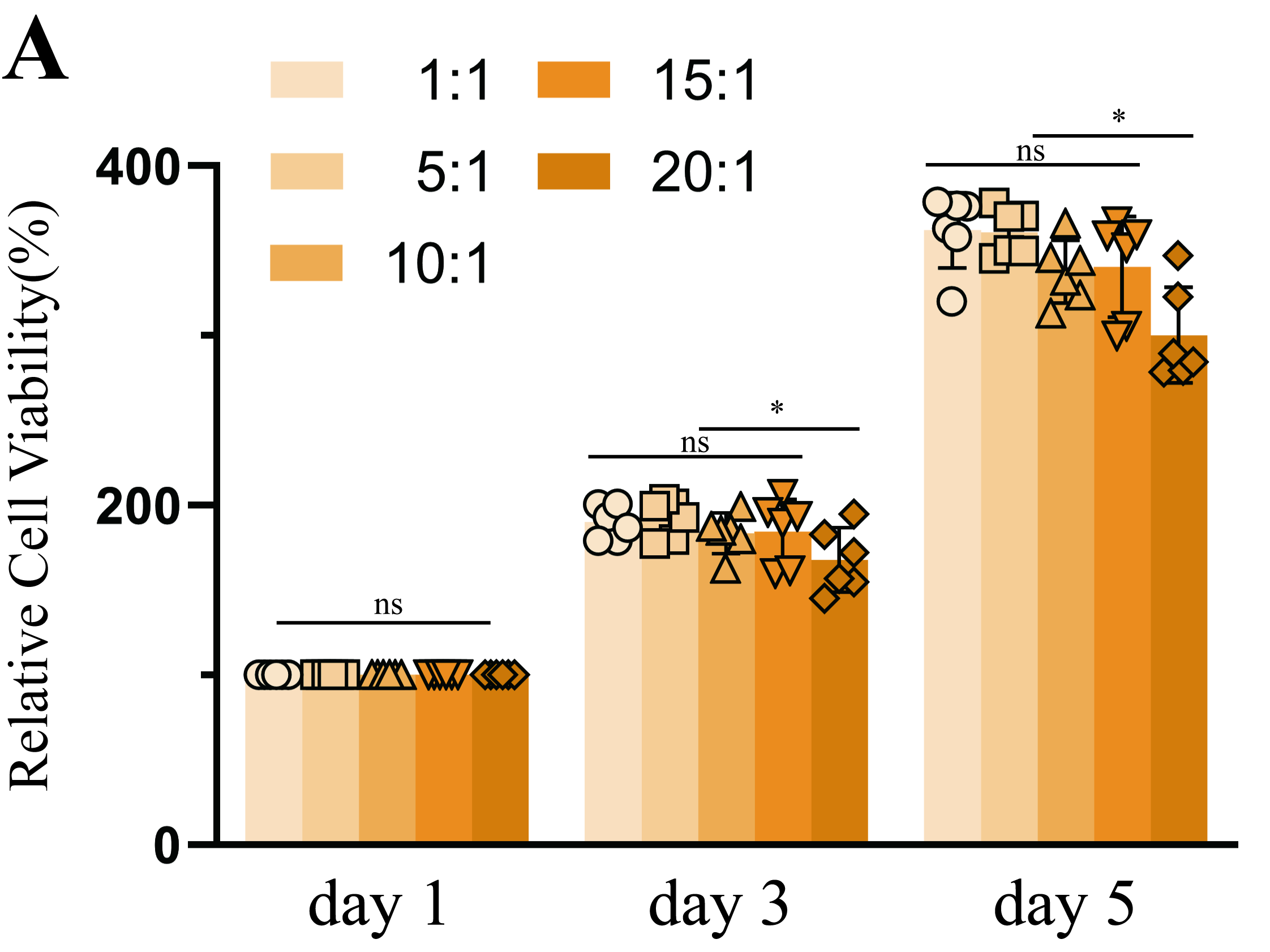


**Supplementary Figure S5.** A) The results of CCK-8 detection of LPS-treated rat NPCs co-cultured with BLNP@GF microspheres (volume ratio of BLNP and GF, 1:1, 5:1, 10:1, 15:1, 20:1) at day 1, 3 and 5 (n = 6). All data were presented as mean ± standard deviation, **p <* 0.05; ***p <* 0.01; ****p <* 0.001; ns, not significant (two-way ANOVA and Tukey’s test compared with each group).


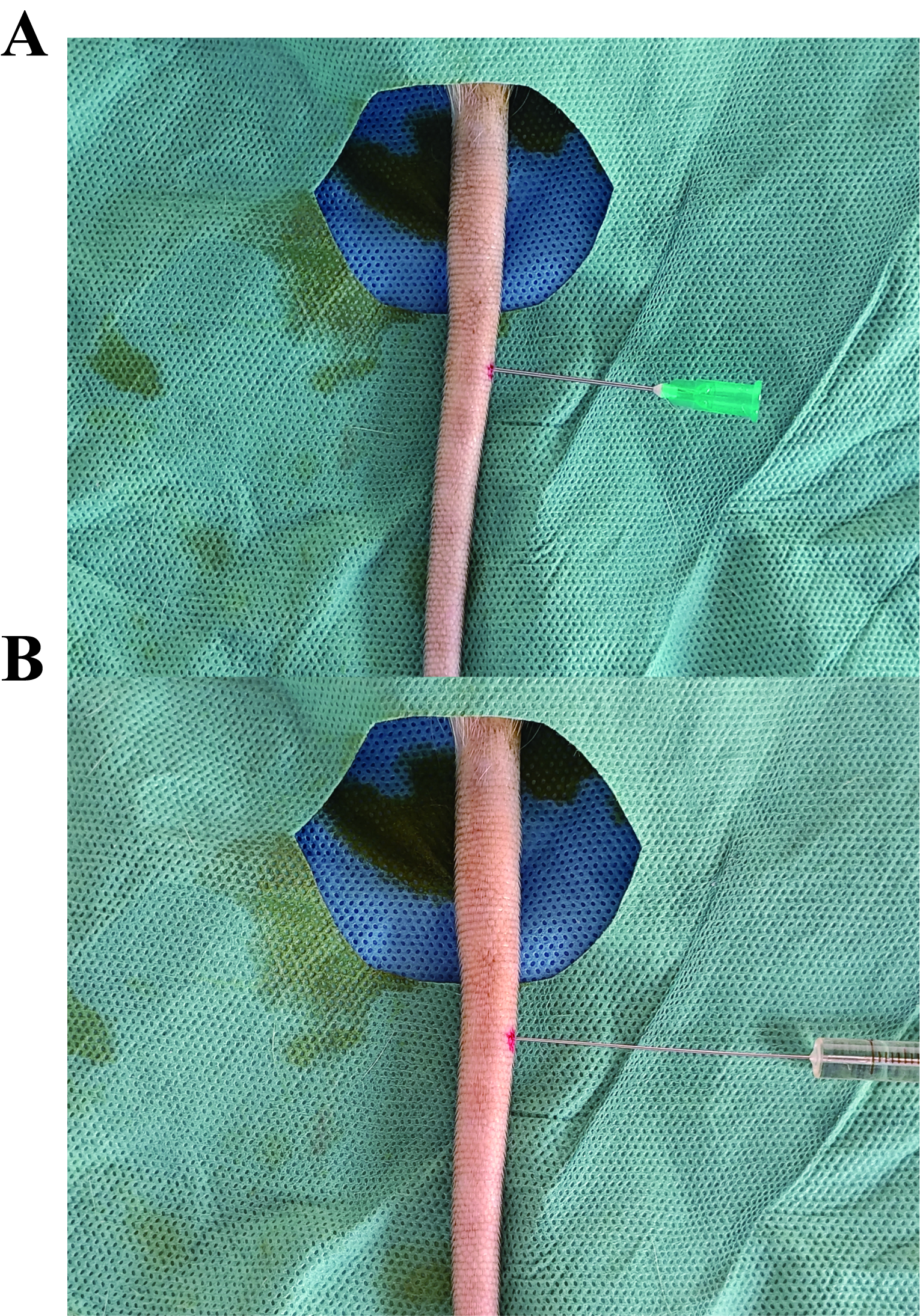


**Supplementary Figure S6. Construction of *in vivo* models.** A) Construction of IVDD model. B) BLNP@GF injectable therapy.
