# Supplementary material for: Brachyury‐Activated Fucoidan Hydrogel Microspheres Rejuvenate Degenerative Intervertebral Discs Microenvironment
Source: Adv Sci (Weinh). 2025 Jun 20;12(34):e04195. doi: 10.1002/advs.202504195 (PMC12442695; doi:10.1002/advs.202504195)
Supplement: Supplementary file 2 — Supporting Information [file ADVS-12-e04195-s002.docx]

**Supplemental Table S1. Antibodies information**

| Antigen | Source | Company | Catalogue | Application | Dilution |
| --- | --- | --- | --- | --- | --- |
| Human-Brachyury | Rabbit | Cell Signaling Technology | 81694 | IHC | 1:200 |
| Human-CA12 | Rabbit | Proteintech | 15180-1-AP | IHC | 1:200 |
| COX2 | Mouse | Proteintech | 66351-1-lg | WB  IF | 1:1000  1:200 |
| iNOS | Rabbit | Abcam | Ab178945 | WB  IF | 1:1000  1:200 |
| Aggrecan | Rabbit | ABclonal | A11691 | WB | 1:1000 |
| Aggrecan | Rabbit | Affinity | DF7561 | IHC | 1:200 |
| Collagen II | Rabbit | Abcam | 34712 | WB  IF | 1:1000  1:200 |
| Rat-Brachyury | Rabbit | Abcam | Ab209665 | WB | 1:1000 |
| IL1-β | Rabbit | Abcam | Ab283818 | IF | 1:200 |
| MMP3 | Rabbit | Abcam | Ab52915 | WB | 1:1000 |
| α-Tubulin | Rabbit | Proteintech | 11224-1-AP | WB | 1:3000 |
| GAPDH | Mouse | Proteintech | 60004-1-Ig | WB | 1:3000 |

**Supplemental Table S2. Sequences of primers**

| Primer name | **Sequences (5’ to 3')** |
| --- | --- |
| Rat-Ptgs2 (COX2) | Forward: 5’-ATTACTGCTGAAGCCCACCC-3'  Reverse: 5’-GGCCCTGGTGTAGTAGGAGA-3' |
| Rat-Nos2 (iNOS) | Forward: 5’-GGAGAAAACCCCAGGTGCTA-3'  Reverse: 5’-GTGGTGAAGGGTGTCGTGAA-3' |
| Rat-TNFα | Forward: 5’-AAATGGCAAATCGGCTGACG-3'  Reverse: 5’-ATCCGAGATGTGGAACTGGC-3' |
| Rat-IL-1β | Forward: 5’-CCTATGTCTTGCCCGTGGAG-3'  Reverse: 5’-CACACACTAGCAGGTCGTCA-3' |
| Rat-brachyury | Forward: 5’-TCCTCAGTTTGGAGGTTCGC-3'  Reverse: 5’-ATTGCTCACAGACCAGAGGC-3' |
| Rat-aggrecan (Acan) | Forward: 5’-AGCCCTTGTCTGAATGGAGC-3'  Reverse: 5’-GTCCACCCCTCCTCACATTG-3' |
| Rat-collagen II (Col2a1) | Forward: 5’-CTCATCGCCACGGTCCTACA-3'  Reverse: 5’-GACGAGGGCTTCCATACATCC-3' |
| Rat-Serpine1 | Forward: 5’-CTTCTTAGAGGCCAGCACCC-3'  Reverse: 5’-ATGTCGTACTCGTGCCCATC-3' |
| Rat-Fgf2 | Forward: 5’-CAAGCGGCTCTACTGCAAGA-3'  Reverse: 5’-CAGCCGTCCATCTTCCTTCA-3' |
| Rat-Ccnd1 | Forward: 5’-GCCATGACTCCCCACGATTT-3'  Reverse: 5’-AGGCAGTCCGGGTCACA-3' |
| Rat-Itga8 | Forward: 5’-GTTCGCTTGTCTCTGTTGCG-3'  Reverse: 5’-CCAGCGAGTAGCCGAAGTAG-3' |
| Rat-Tgfb2 | Forward: 5’-TCCCCTCCGAAAATGCCATC-3'  Reverse: 5’-AATCCGTTGTTCAGCCACTCT-3' |
| Rat-β-actin | Forward: 5’-CCCTAAGGCCAACCGTGAA-3'  Reverse: 5’-CACGCACGATTTCCCTCTCA-3' |
